# Herbal Medicine Compared to Placebo for Chronic Obstructive Pulmonary Disease: A Systematic Review and Meta-Analysis

**DOI:** 10.3389/fphar.2021.717570

**Published:** 2021-10-20

**Authors:** Chan-Young Kwon, Boram Lee, Beom-Joon Lee, Kwan-Il Kim, Hee-Jae Jung

**Affiliations:** ^1^ Department of Oriental Neuropsychiatry, Dong-eui University College of Korean Medicine, Busan, South Korea; ^2^ Department of Clinical Korean Medicine, Graduate School, Kyung Hee University, Seoul, South Korea; ^3^ Division of Allergy, Immune and Respiratory System, Department of Internal Medicine, College of Korean Medicine, Kyung Hee University, Seoul, South Korea

**Keywords:** COPD, east asian traditional medicine, chronic bronchitis, emphysema, systematic review

## Abstract

Chronic obstructive pulmonary disease (COPD) is a respiratory disease characterized by irreversible airflow limitation. Many COPD patients use complementary and alternative modalities, including herbal medicines (HMs). This systematic review investigated the effectiveness and safety of HM in managing COPD symptoms compared to placebo. Nine electronic databases were searched to identify relevant randomized controlled trials (RCTs) up to February 12, 2021. The Cochrane risk of bias tool was used to assess the methodological qualities of the included studies. Primary outcomes were lung function parameters and exercise capacity. A meta-analysis was conducted to determine the effect size for homogeneous outcomes. Fourteen studies were included. There was low to very low quality evidence that HM significantly improved forced expiratory volume in 1  s (FEV1) (L), FEV1 (%) and 6-minute walk distance, as well as moderate quality evidence that HM significantly improved forced vital capacity (FVC) (L) compared to placebo. However, according to low quality evidence, there was no significant difference in FEV1/FVC (%) or vital capacity (L) between the groups. Low to moderate evidence suggests that HM has the potential to help improve some respiratory functions, COPD symptoms, and some aspects of quality of life in COPD patients compared to placebo. However, these findings are challenged by the poor methodological quality of the included studies, the heterogeneity of HMs used, and potential publication bias. Therefore, the findings could be significantly influenced by further larger, more rigorous RCTs on this topic. Moreover, it may also be recommended to develop standardized HMs focused on some individual herbs that are frequently used or expected to play an important role in patients with COPD, and to elucidate the underlying mechanisms.

## Introduction

Chronic obstructive pulmonary disease (COPD), including chronic bronchitis and emphysema, is a respiratory disease characterized by irreversible airflow limitation that causes symptoms like chronic cough, phlegm, and dyspnea (Singh et al., 2019). COPD is relatively more common in men than in women, and its prevalence is estimated to be approximately 16% in men and 10% in women worldwide (Varmaghani et al., 2019). The most common risk factor for COPD is smoking, with toxic chemicals, air pollution, and chronic bronchitis being the other causes contributing to damage to the airways and lung parenchyma by chronic inflammation (Singh et al., 2019). In addition, older age, being underweight or obese, and low economic and social status are considered to be relevant risk factors (Osman et al., 2017).

Treatment of COPD can be broadly classified into the initial and follow-up phases (Singh et al., 2019). Pharmacological treatments include long-acting bronchodilators, long-acting muscarinic antagonists, inhaled corticosteroids, long-acting β2-agonist, or their combination, depending on respiratory ability and frequency of exacerbation (Singh et al., 2019). Moreover, quitting smoking is an important lifestyle modification to prevent exacerbations in COPD patients (Ambrosino and Bertella, 2018). Comorbidities, such as cardiovascular disease, osteoporosis, poor sleep quality, and depression, can adversely affect the health of COPD patients; therefore, these conditions should be treated concurrently (Recio Iglesias et al., 2020).

The main treatment for COPD involves bronchodilators, which can improve patients’ respiratory symptoms and quality of life, and prevent exacerbation; however, this therapeutic strategy alone is not effective enough and does not cure the underlying etiology. Therefore, pulmonary rehabilitation, long-term oxygen therapy, and phosphodiesterase-4 inhibitors be additionally used (Candela et al., 2019). In this respect, herbal medicine (HM) can also be considered an effective adjuvant therapy for COPD. The various active ingredients contained in HM, particularly flavonoid derivatives, have the potential to exert therapeutic effects on the underlying pathology of COPD by reducing inflammation in the lungs (Kim et al., 2017). Moreover, a systematic review published in 2014 concluded that HM can improve the clinical symptoms and quality of life of patients with COPD based on 15 high quality studies (Chen et al., 2014). However, evidence in this area is not yet established, and the authors conclude that, in particular, double-blind randomized controlled trials (RCTs) are needed (Chen et al., 2014).

A well-designed placebo control is needed for successful double-blind RCTs using HM. Unlike chemical drugs, HM can be described by its characteristics of taste and aroma, which poses a challenge for successful blinding of participants. However, there has not yet been a comprehensive review of the effectiveness and safety of HM for COPD compared to placebo from this perspective. Therefore, this systematic review and meta-analysis was conducted to investigate the effectiveness and safety of HM in managing COPD symptoms compared to placebo.

## Methods

The protocol of this study was pre-registered with OSF registries (10.17605/OSF.IO/3EJD2). This systematic review complied with the Preferred Reporting Items for Systematic Reviews and Meta-Analyses (PRISMA) 2010 statement (Supplementary File S1) (Moher et al., 2009).

### Data Sources and Search Strategy

One researcher (BL) searched nine electronic databases, including three core ones (Medline via PubMed, EMBASE *via* Elsevier, and Cochrane Central Register of Controlled Trials), one database related to complementary and alternative medicine (Allied and Complementary Medicine Database), two Korean databases (Korean Studies Information Service System and Korea Citation Index), and three Chinese databases (China National Knowledge Database, WanFang data, and VIP). The search date was February 12, 2021. All relevant studies published up to the search date were considered without language and publication status limitations. Grey literature consisting of dissertations and conference papers was also included. In addition, the bibliographic list of the relevant articles was reviewed and missing data related to the potentially relevant studies were collected through manual searches on Google Scholar. Finally, the search strategy was confirmed through sufficient discussions with experts on systematic reviews and clinical experts on COPD. The search strategies and results from each database are listed in Supplementary File S2.

### Eligibility Criteria

The inclusion criteria for this review were as follows. **1) Type of study:** Only RCTs were included in this review. **2) Types of participants:** Adult patients (over 18 years of age) diagnosed with COPD were included, regardless of sex, COPD stage, and status of exacerbations. Patients with COPD and other pathological conditions were excluded from the study. Studies including people with COPD and other respiratory diseases (e.g., asthma COPD overlap syndrome) were also excluded. **3) Types of interventions:** Only oral HM based on East Asian traditional medicine (EATM) theory was considered. There were no restrictions on the dosage form of HM. It was allowed when usual care or other active interventions for COPD were applied to the treatment and control groups at the same time. Studies that used a single herb or an ingredient extracted from one herb as their intervention or used HM not based on the EATM theory were excluded. **4) Types of controls:** We only allowed oral placebo HM as a control. In this review, placebo HM refers to a substance in which the difference in taste, aroma, and texture from active HM cannot be discerned. Studies that did not describe a similarity between placebo and original HM in taste or aroma as well as visual characteristics were excluded. **5) Types of outcomes:** Primary outcomes included lung function parameters (e.g., forced expiratory volume in 1 s [FEV1], forced vital capacity [FVC], FEV1/FVC, or vital capacity [VC]) and exercise capacity (6-min walk distance [6MWD]). Secondary outcomes included severity of dyspnea (e.g., the modified Medical Research Council [mMRC] dyspnea scale, or other assessment tools such as patient-reported measures, self-assessment, questionnaires, etc.), quality of life (COPD assessment test [CAT], other assessment tools such as the St. George respiratory questionnaire [SGRQ]), and adverse events or safety measurements. Studies that did not report outcomes of interest, especially those that did not report respiratory function or respiratory symptoms, were excluded.

### Study Selection

After removing duplicate studies, two researchers (CYK and BL) independently screened the titles and abstracts of the searched studies to check for eligibility. Then, the full-texts of the screened studies were reviewed by two researchers (CYK and BL) for inclusion in this review. Discrepancies were resolved by discussion with a third researcher (KIK). EndNote X8 (Clarivate Analytics, Philadelphia, United States ) was used to manage the bibliographic information.

### Data Extraction

The extracted data from the eligible studies by two researchers (CYK and BL) were entered into a pre-defined Microsoft Excel file. Microsoft Excel 2016 (Microsoft, Redmond, WA, United States ) was used for the data extraction. The following items were extracted: name of the first author, publication year, publication country, study setting, sample size and withdrawals, details of participants, details of treatment and control interventions, outcome measures, safety data, information for assessment of the risk of bias, and funding sources. Discrepancies were resolved by discussion of the researchers (CYK and BL), or if needed, with a third researcher (KIK).

### Risk of Bias Assessment

The Cochrane risk of bias tool was used to assess the methodological quality of the included RCTs. In this tool, the methodological quality of RCTs is evaluated in terms of risk of bias, including random sequence generation, allocation concealment, blinding of participants, personnel and outcome assessors, completeness of outcome data, selective reporting, and other biases (Higgins et al., 2011). Each bias item is evaluated as “low risk,” “unclear risk,” or “high risk” (10). Quality assessment was performed by two independent researchers (CYK and BL). Discrepancies were resolved by discussion with a third researcher (KIK).

### Data Analysis and Synthesis

All included studies were descriptively analyzed. If enough homogeneous data existed, meta-analysis was performed using RevMan 5.4 (The Cochrane Collaboration, London, England). Dichotomous data were presented as risk ratios (RRs) with 95% confidence intervals (CIs) while continuous data were reported as mean difference (MD) with 95% CIs. Heterogeneity between the studies in terms of effect measures was assessed using both the χ^2^ test and the *I* ^*2*^ statistic. *I* ^*2*^ values ≥50% and ≥75% were considered indicative of substantial and considerable heterogeneity, respectively. Meta-analysis was performed using a random-effects model if the included studies had significant heterogeneity (*I* ^*2*^ value ≥50%). When the heterogeneity was not significant or the number of studies included in the meta-analysis was very small, in which the estimate of the between-study variance had poor precision, a fixed-effect model was used (Graves, 2002). Subgroup analysis was conducted by classifying COPD patients according to the condition, including stable COPD, acute exacerbation of COPD (AECOPD), and treatment duration (≤ 4 weeks or ≥2 months). In addition, a sensitivity analysis was conducted, excluding studies that did not properly conduct the blinding of participants and personnel.

### Dealing With Missing Data

The authors contacted the corresponding author via email regarding unclear information in the study. If data were still insufficient after contacting the corresponding author or if contact was impossible, only the available data were included in the analysis.

### Publication Bias

If the number of studies included in the meta-analysis was 10 or more, a funnel plot was used to assess potential publication bias.

### Quality of Evidence

Two researchers (CYK and BL) assessed the quality of evidence for the main findings using the Grading of Recommendations, Assessment, Development and Evaluations (GRADE) approach (Balshem et al., 2011). The risk of bias of the studies included in each meta-analysis, imprecision, inconsistency, indirectness of the results, and relevant publication bias were evaluated for each finding. The quality of evidence is categorized into four categories, as follows: “high,” “moderate,” “low,” or “very low.”

## Results

### Study Selection

**FIGURE 1:**
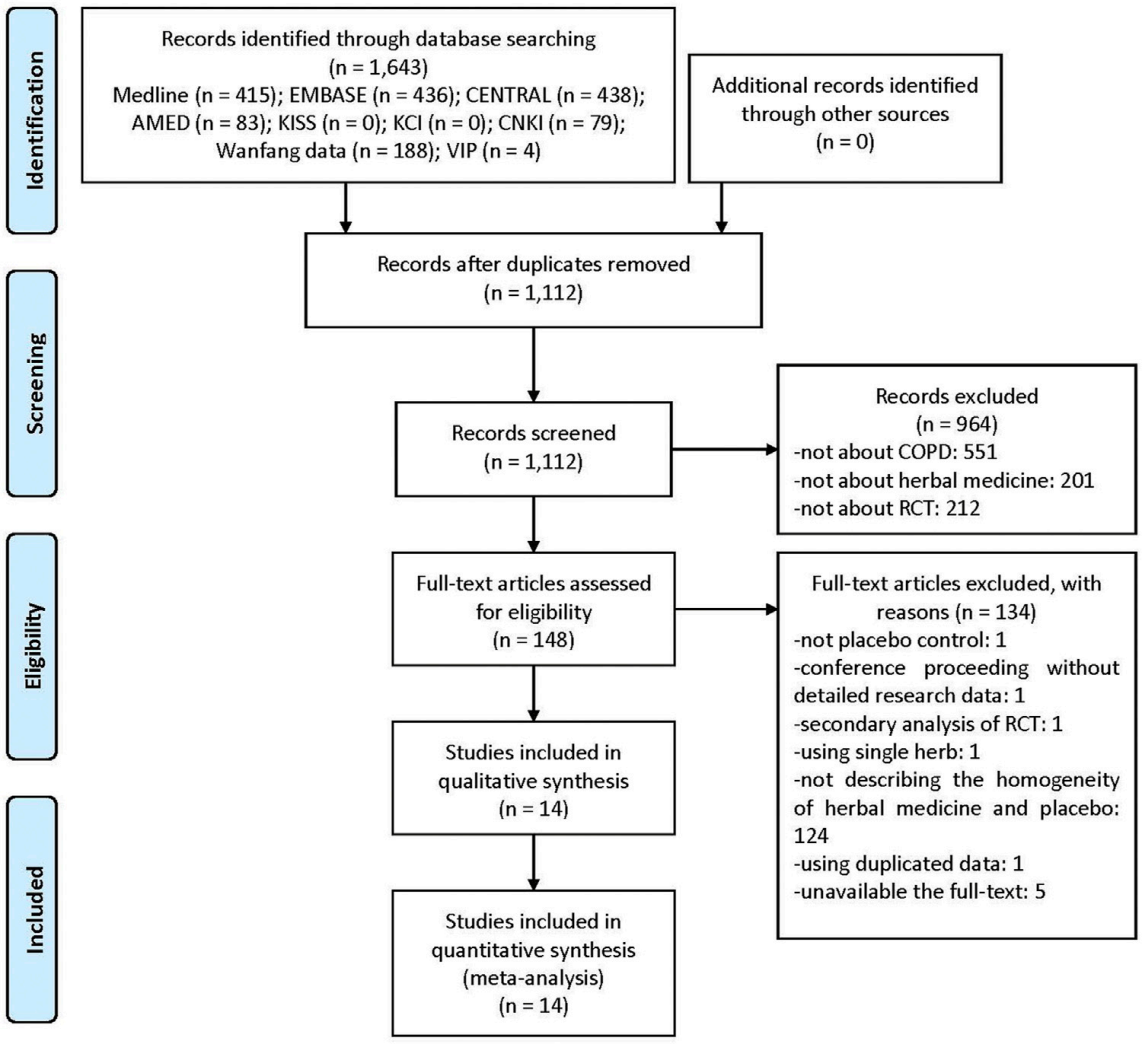
A PRISMA flow diagram of the literature screening and selection process. AMED, Allied and Complementary Medicine Database; CENTRAL, Cochrane Central Register of Controlled Trials; CNKI, China National Knowledge Infrastructure; COPD, chronic obstructive pulmonary disease; KCI, Korea Citation Index; KISS, Koreanstudies Information Service System; RCT, randomized controlled trial.

Of the 1,643 documents searched initially, titles and abstracts of 1,112 were screened, excluding duplications. Consequently, 148 potentially relevant papers were selected. Upon reviewing the full-text of these studies, one RCT not using placebo, one conference proceeding without detailed research data, one secondary analysis of RCT, one using a single herb rather than complex HM, 124 with unclear differences between active HM and placebo without obvious differences in taste or aroma (e.g., using caramel placebo), one using duplicated data, and five with unavailable full-texts were excluded (Supplementary File S3). Finally, 14 RCTs were included in this systematic review and meta-analysis (Figure 1) (Li et al., 2011; Liu, 2011; Guo et al., 2014; Liu et al., 2014; Wang et al., 2014; Chen, 2015; Shi, 2016; Gao et al., 2017; Hong, 2018; Huang et al., 2019; Jin et al., 2019; Luo et al., 2019; Hu et al., 2020).

### Study Characteristics

The included studies were published between 2011 and 2020, all of which were conducted in China. Eight (Liu, 2011; Sun, 2013; Guo et al., 2014; Wang et al., 2014; Shi, 2016; Hong, 2018; Jin et al., 2019; Hu et al., 2020) involved patients with stable COPD, while four (Li et al., 2011; Liu et al., 2014; Huang et al., 2019; Luo et al., 2019) involved patients with AECOPD. For the other two RCTs (Chen, 2015; Gao et al., 2017), the subjects’ COPD status were unclear. In one study, Wang et al. (2014) compared two HMs and one placebo in a three-arm clinical trial. Therefore, we separated and extracted the effects of the two HMs used in this study into Wang 2014(A) and Wang 2014(B). In all studies, the study setting was a hospital, and eight studies (Li et al., 2011; Liu, 2011; Guo et al., 2014; Liu et al., 2014; Wang et al., 2014; Chen, 2015; Huang et al., 2019; Jin et al., 2019; Luo et al., 2019; Hu et al., 2020) that identified the source of funding were supported by national or provincial funding. All studies performed conventional therapies, such as health education and Western medication therapy, including inhaled albuterol and long-acting bronchodilators, to all participants. Eight studies (Guo et al., 2014; Liu et al., 2014; Wang et al., 2014; Chen, 2015; Hong, 2018; Huang et al., 2019; Jin et al., 2019; Hu et al., 2020) were approved by the institutional review board prior to study initiation, and 12 studies (Liu, 2011; Sun, 2013; Guo et al., 2014; Liu et al., 2014; Wang et al., 2014; Chen, 2015; Shi, 2016; Hong, 2018; Huang et al., 2019; Jin et al., 2019; Luo et al., 2019; Hu et al., 2020) received informed consent from participants. The published articles did not confirm whether other studies were approved by the institutional review board or an informed consent was received from the participants. Twelve studies (Sun, 2013; Guo et al., 2014; Liu et al., 2014; Wang et al., 2014; Chen, 2015; Shi, 2016; Gao et al., 2017; Hong, 2018; Huang et al., 2019; Jin et al., 2019; Luo et al., 2019) recruited participants according to specific pattern identification, of which deficiency of specific organs was the most common, seen in five studies (Sun, 2013; Wang et al., 2014; Chen, 2015; Shi, 2016; Jin et al., 2019); phlegm-heat or phlegm-turbidity obstructing the lungs in three studies (Liu et al., 2014; Gao et al., 2017; Luo et al., 2019); blood stasis in two studies (Li et al., 2011; Huang et al., 2019); and deficiency of the lung, spleen, and kidney with retention of phlegm and blood stasis in two studies (Guo et al., 2014; Hong, 2018). Nine studies (Liu, 2011; Sun, 2013; Guo et al., 2014; Liu et al., 2014; Wang et al., 2014; Chen, 2015; Shi, 2016; Hong, 2018; Hu et al., 2020) reported the occurrence of adverse events during the clinical trial period. Detailed study characteristics of the included studies are summarized in Supplementary Table S1 **.**

### Details of HM Used

A wide variety of HMs were used in the included studies. With regard to dosage type, decoction was the most common in six studies (Sun, 2013; Chen, 2015; Shi, 2016; Gao et al., 2017; Huang et al., 2019; Jin et al., 2019), followed by granules in five studies (Li et al., 2011; Liu, 2011; Guo et al., 2014; Liu et al., 2014; Hong, 2018). In general, *Poria cocos (Schw.) Wolf [*Polyporaceae*; Poria(Hoelen)*] and *Citrus unshiu Markovich [*Rutaceae*; Citri unshius pericarpium]* were the most frequently used herbs in six studies, followed by *Codonopsis pilosulae (Fr.) Nannf [*Campanulaceae*; Codonopsis pilosulae radix]*, *Atractylodes macrocepha-la Koidz [*Asteraceae*; Atractylodis rhizoma alba]*, and *Glycyrrhiza uralensis Fisch [*Leguminosae*; Glycyrrhizae radix]* in five studies each. To evaluate stable COPD, *Codonopsis pilosulae (Fr.) Nannf [*Campanulaceae*; Codonopsis pilosulae radix]* was used the most in five studies, followed by *Atractylodes macrocepha-la Koidz [*Asteraceae*; Atractylodis rhizoma alba]*, *Poria cocos (Schw.) Wolf [*Polyporaceae*; Poria(Hoelen)*], *Cornus officinalis Sieb. et Zucc [*Cornaceae*; Corni fructus]*, *Rehmannia glutinosa (Gaertner) Libosch [*Scrophulariaceae*; Rehmanniae radix preparat]*, *Citrus unshiu Markovich [*Rutaceae*; Citri unshius pericarpium]*, and *Astragalus membranaceus Bunge [*Leguminosae*; Astragali radix]* in three studies each. Herbs were not commonly used in more than three studies on AECOPD. Instead, *Paeonia lactiflora Pallas [*Paeoniaceae*; Paeoniae radix]*, *Prunus persica (L.) Batsch [*Rosaceae*; Persicae semen]*, *Rheum palmatum L [*Polygonaceae*; Rhei radix et rhizoma]*, *Prunus armeniaca L.* var. *ansu Maxim [*Rosaceae*; Armeniacae semen]*, *Pinellia ternata (Thunb.) Breit [*Araceae*; Pinelliae rhizoma]*, *Poria cocos (Schw.) Wolf [*Polyporaceae*; Poria(Hoelen)*], and *Citrus unshiu Markovich [*Rutaceae*; Citri unshius pericarpium]* were used in two studies. In three studies (Li et al., 2011; Chen, 2015; Gao et al., 2017), herbs were added to the basic HM prescription according to specific symptoms or pattern identification. The period of HM administration varied widely from day 10–180. In particular, studies on AECOPD had relatively short treatment periods ranging from 10 to 14 days. Four studies (Liu, 2011; Guo et al., 2014; Wang et al., 2014; Hu et al., 2020) performed follow-up after the completion of HM administration, and the duration varied from 4 months to 1 year **(** Supplementary Table S2).

### Risk of Bias Assessment

**FIGURE 2:**
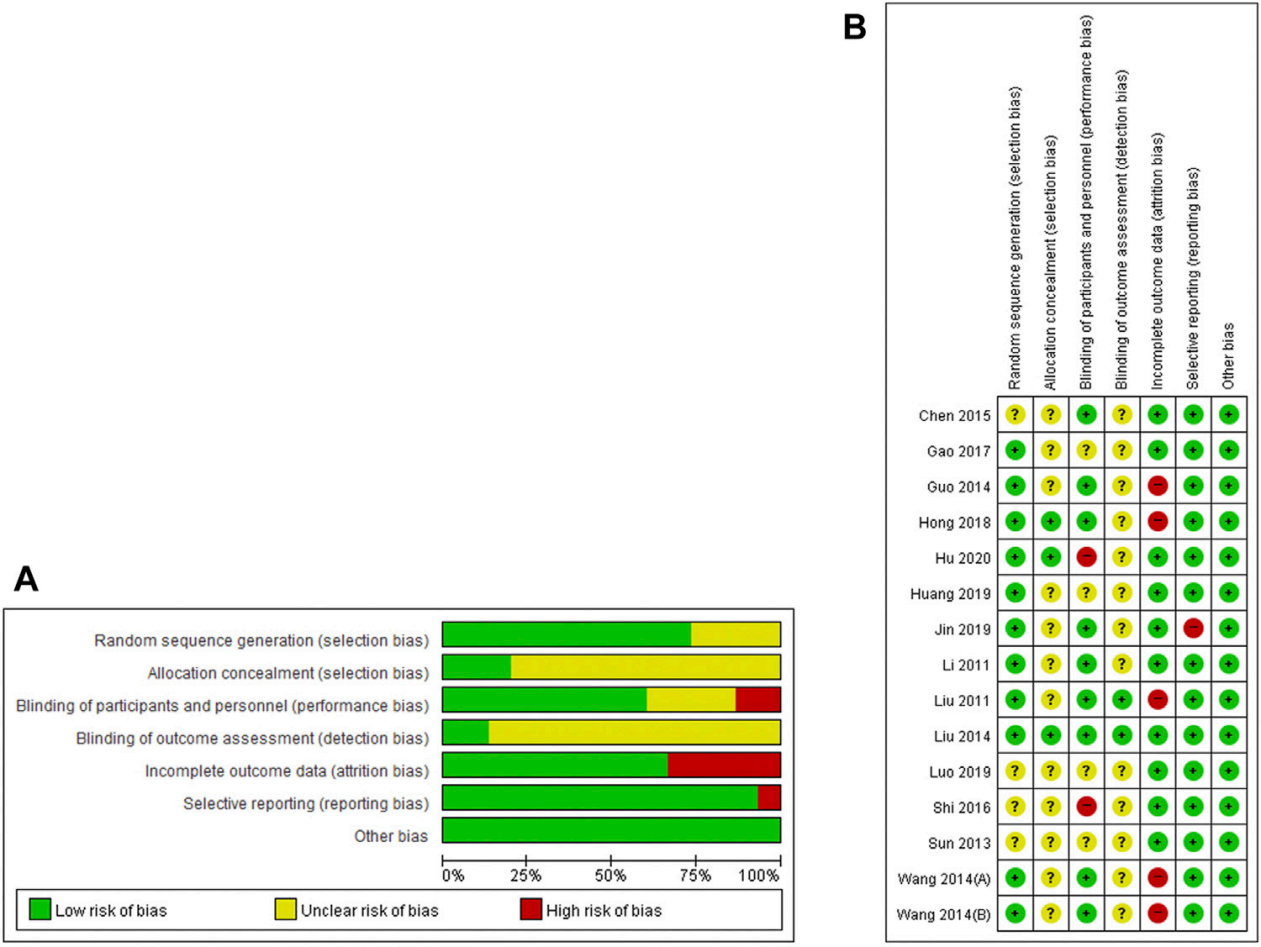
Risk of bias for all included studies. Low, unclear, and high risk, respectively, are represented with the following symbols: “+,” “?” and “−”.

Ten studies (Li et al., 2011; Liu, 2011; Guo et al., 2014; Liu et al., 2014; Wang et al., 2014; Gao et al., 2017; Hong, 2018; Huang et al., 2019; Jin et al., 2019; Hu et al., 2020) using appropriate random number generation methods, such as random number tables, were evaluated as having a low risk of bias in the random sequence generation domain. Three studies (Liu et al., 2014; Hong, 2018; Hu et al., 2020) using opaque sealed envelopes to conceal allocation were evaluated as having a low risk of bias in the allocation concealment domain. Eight (Li et al., 2011; Liu, 2011; Guo et al., 2014; Liu et al., 2014; Wang et al.; Chen, 2015; Hong, 2018; Jin et al., 2019) and two studies (Liu, 2011; Liu et al., 2014) properly blinded participants and personnel as well as outcome assessors, respectively. Four studies (Liu, 2011; Guo et al., 2014; Wang et al., 2014; Hong, 2018) that performed only per-protocol analysis were evaluated as having a high risk of attrition bias, and one study (Jin et al., 2019) that presented only the total effective rate (TER) without raw data was evaluated as having a high risk of reporting bias **(** Figure 2 **)**.

### Effectiveness and Safety of HM for COPD

#### Lung Function (Primary Outcome)

**FIGURE 3:**
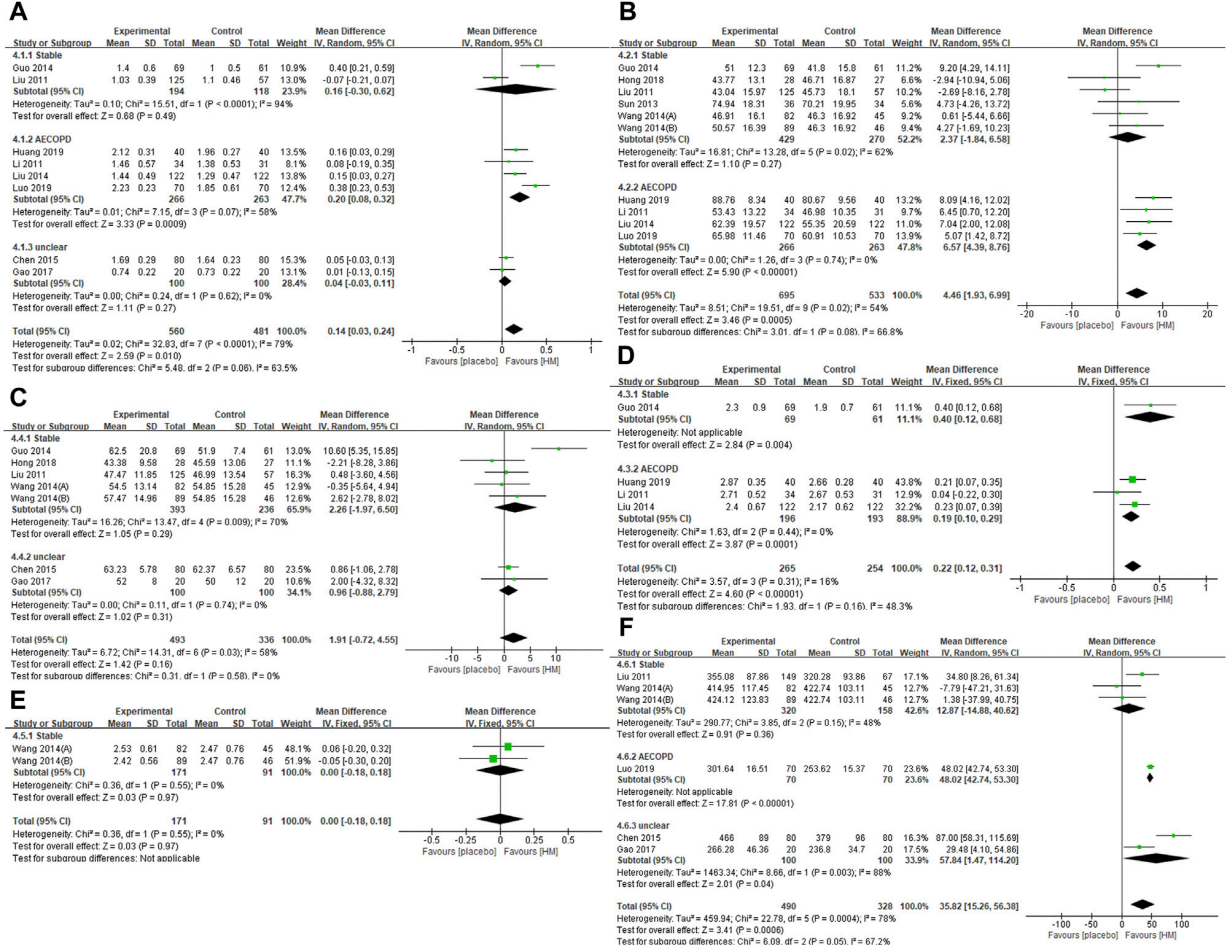
Forest plots of **(A)** FEV1 (L), **(B)** FEV1 (%), **(C)** FEV1/FVC (%), **(D)** FVC (L), **(E)** VC (L), and **(F)** 6MWD. AECOPD, acute exacerbations of chronic obstructive pulmonary disease; FEV1, forced expiratory volume in one second; FVC, forced vital capacity; VC, vital capacity; 6MWD, 6-min walking distance.

Post-treatment, there were significant differences between the HM and placebo groups in FEV1 (L) (eight studies; MD 0.14, 95% CI 0.03 to 0.24; *I* ^*2*^ = 79%), FEV1 (%) (10 studies; MD 4.46, 95% CI 1.93 to 6.99; *I* ^*2*^ = 54%), and FVC (L) (four studies; MD 0.22, 95% CI 0.12 to 0.31; *I* ^*2*^ = 16%). In subgroup analysis, however, the significant benefits of HM were observed in FEV1 (L) and FEV1 (%) only for AECOPD when the treatment duration was ≤ 4 weeks. They were not observed for stable COPD when the treatment duration was ≥2 months. The significant benefits of FVC (L) were maintained according to the COPD status and treatment duration, regardless of the subgroup. In FEV1/FVC (%) (seven studies; MD 1.91, 95% CI −0.72 to 4.55; *I* ^*2*^ = 58%), and VC (L) (two studies; MD 0.00, 95% CI −0.18 to 0.18; *I* ^*2*^ = 0%), significant difference was not observed according to COPD status and treatment duration, regardless of the subgroup. In the sensitivity analysis excluding studies not properly conducting blinding of participants and personnel, the significant effect on FEV1 (L) (five studies; MD 0.11, 95% CI −0.02 to 0.24; *I* ^*2*^ = 77%) and FEV1 (%) (seven studies; MD 3.49, 95% CI −0.10 to 7.08; *I* ^*2*^ = 63%) disappeared, and the results of the remaining outcomes were not significantly affected (Supplementary Table S3) (Figures 3A–E).

#### Exercise Capacity (Primary Outcome)

At post-treatment, HM group showed significantly better 6MWD (m) (six studies; MD 35.82, 95% CI 15.26 to 56.38; *I* ^*2*^ = 78%). In subgroup analysis, however, the significant benefits of 6MWD of HM remained for AECOPD and unclear COPD when the treatment duration was ≤ 4 weeks, but not for stable COPD when the treatment duration was ≥2 months. In the sensitivity analysis excluding studies not properly conducting blinding of participants and personnel, the significant effect on 6MWD remained (four studies; MD 38.48, 95% CI 22.52 to 54.45; *I* ^*2*^ = 85%) (Supplementary Table S3) (Figure 3F).

#### Symptom Severity (Secondary Outcome)

At post-treatment, the HM group showed significantly lower mMRC scores (two studies; MD −1.13, 95% CI, −1.21 to −1.05; *I* ^*2*^ = 94%). In subgroup analysis, however, the significant benefits on the mMRC score of HM remained for AECOPD, but not for stable COPD. In stable COPD patients, the frequency of acute exacerbation at the 1-year follow-up was significantly lower in the HM group than in the placebo group (two studies; MD −0.60, 95% CI −0.69 to −0.51; *I* ^*2*^ = 0%). In the sensitivity analysis, excluding studies not properly conducting blinding of participants and personnel, the significant effect on the frequency of acute exacerbation in the 1-year follow-up remained (one study; MD −0.60, 95% CI −0.69 to −0.51). The HM group showed significantly higher TER based on clinical symptoms (10 studies; RR 1.26, 95% CI 1.11 to 1.44; *I* ^*2*^ = 76%). The significant benefits of TER were maintained according to COPD status and treatment duration, regardless of the subgroup. However, in the sensitivity analysis excluding studies not properly conducting blinding of participants and personnel, the significant effect of HM on TER disappeared on stable COPD (three studies; RR 1.80, 95% CI 0.88 to 3.65; *I* ^*2*^ = 95%) and AECOPD (one study; RR 1.08, 95% CI 0.92–1.27) (Supplementary Table S3).

#### Quality of Life (Secondary Outcome)

Post-treatment, the HM group showed significantly lower CAT scores (five studies; MD −3.78, 95% CI −5.73 to −1.83; *I* ^*2*^ = 78%) and SGRQ scores (six studies; MD −7.56, 95% CI −14.4 to −0.72; *I* ^*2*^ = 93%) than the placebo group. In the subgroup analysis, however, the significant benefits on CAT score of HM remained for stable COPD and AECOPD, but not for unclear COPD. In addition, the significant benefits of the SGRQ score of HM were not retained for both stable COPD and AECOPD. In the World Health Organization quality of life instruments-abbreviated version (WHOQOL-BREF), the psychological domain was significantly higher in the HM group (three studies; MD 0.99, 95% CI 0.35 to 1.64; *I* ^*2*^ = 66%). Subgroup analysis according to COPD status and treatment duration showed a significant difference between groups only in AECOPD when the treatment duration was ≤ 4 weeks, but not in stable COPD when the treatment duration was ≥2 months. The social relationships domain of WHOQOL-BREF was significantly lower in the HM group (one study; MD −0.83, 95% CI −1.55 to −0.11). However, there were no differences in physical health (one study; MD 0.46, 95% CI −0.23 to 1.15) and in the environment domain (one study; MD −0.04, 95% CI −0.72 to 0.64). There was no significant difference detected in the sensitivity analysis excluding studies that did not properly conduct blinding of participants and personnel (Supplementary Table S3).

#### Safety Data

There were no significant differences between the HM and placebo groups in the incidence of adverse events (eight studies; RR 1.08, 95% CI 0.51 to 2.28; *I* ^*2*^ = 22%). This non-significance was maintained according to COPD status and treatment duration, regardless of the subgroup. In addition, in the sensitivity analysis excluding studies that did not properly conduct blinding of participants and personnel, this non-significance still remained (Supplementary Table S3). The adverse events reported in the HM group were cold limbs (1 case), lip color turning red (1 case), glycosuria (1 case), diarrhea (1 case), and proteinuria (1 case) in one study (Liu, 2011) and sweating (1 case) in another study (Shi, 2016). Hong (2018) reported a case in the HM group in which the color of the sputum changed from thick white to light yellow. Two studies (Liu et al., 2014; Hu et al., 2020) reported adverse reactions in six cases and diarrhea in two cases in the HM group.

### Impact of HM on Inflammation-Related Parameters

Despite the lack of pre-defined outcomes of interest, some studies have reported an effect of HM on inflammation-related parameters in COPD patients. A study by Guo et al. (2014), in which patients with stable COPD were administered Bufei granules for 12 weeks reported that serum interleukin (IL)-6, IL-8, tumor necrosis factor (TNF)-α, and transforming growth factor (TGF)-β1 after treatment were not significantly different between the two groups (*p* > 0.05). However, IL-8, TNF-α, and TGF-β1 decreased significantly only in the HM group (*p* < 0.05). A study by Sun (2013), in which patients with stable COPD were administered Sijunzi-tang combined with Jinkui Shenqi pills for 4 weeks, serum IL-8 levels in the HM group after treatment were significantly lower than those in the placebo group (*p* < 0.05). However, there was no significant difference in serum TNF-α levels between the groups (*p* > 0.05). In a study by Wang et al. (2014), in which patients with stable COPD were administered Bushen Yiqi granules or Bushen Fangchaun tablets for 180 days, serum IL-17 levels in the two HM groups were significantly lower than those in the placebo group (both, *p* < 0.05). However, there were no significant differences in other parameters, including serum IL-6, IL-8, IL-10, IL-1β, TGF-β1, TNF-α, and cortisol between groups (all, *p* > 0.05). In a study by Huang et al. (2019), in which patients with AECOPD were administered Mengshiguntan-wan for 2 weeks, levels of IL-17 and 8-iso-prostaglandins in exhaled breath condensates were significantly lower than those in the placebo group (both, *p* < 0.05), while the levels of IL-10 in exhaled breath condensates were significantly higher than those in the placebo group (*p* < 0.05).

### Publication Bias

**FIGURE 4:**
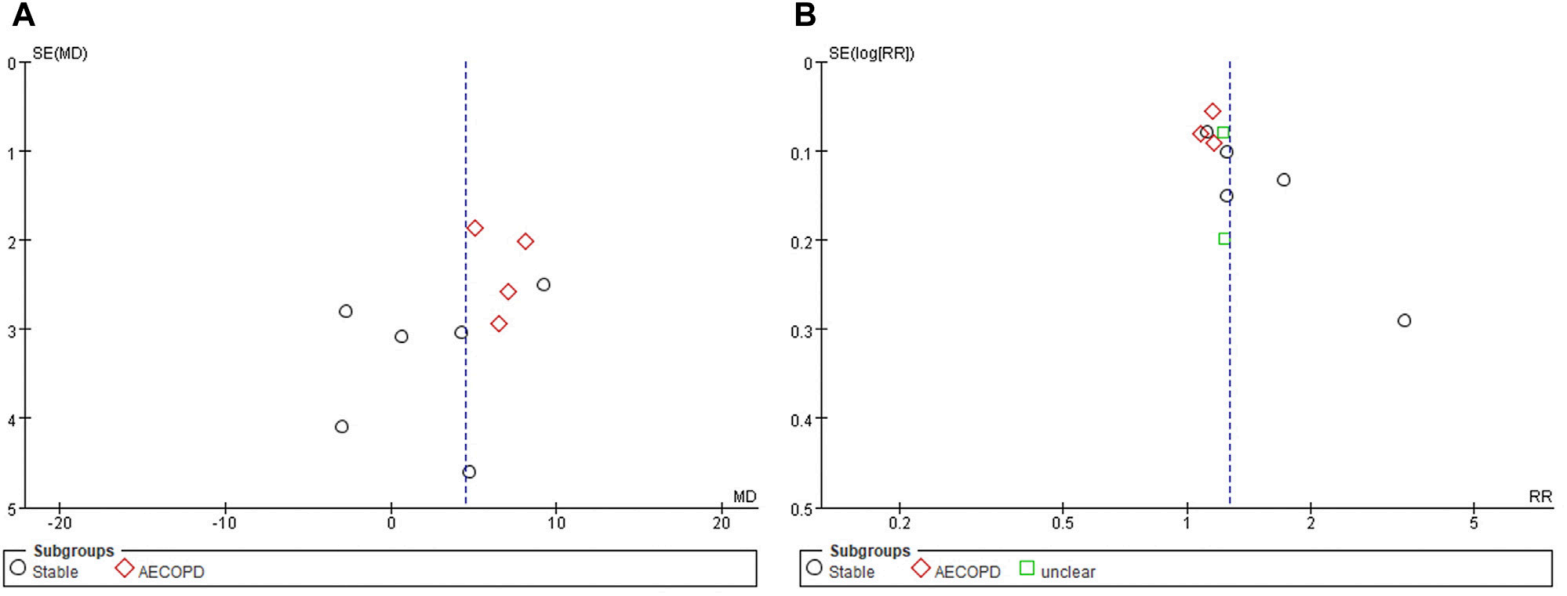
Funnel plots of **(A)** FEV1 (%) and **(B)** TER based on clinical symptoms. AECOPD, acute exacerbations of chronic obstructive pulmonary disease; FEV1, forced expiratory volume in one second; TER, total effective rate.

Funnel plot evaluation was possible only for FEV1 (%) and TER based on clinical symptoms. All of them showed obvious asymmetries, suggesting a potential publication bias (Figures 4A,B).

### Quality of Evidence

The quality of evidence for the main results was mostly “very low” to “moderate”. The reason for downgrading was the high risk of bias of the included studies, inconsistency of results due to high heterogeneity, and imprecision of results due to wide CIs and small sample sizes (Supplementary Table S3).

## Discussion

### Summary of Evidence

This systematic review comprehensively evaluated the efficacy and safety of HM in COPD compared to placebo. A total of 14 placebo-controlled RCTs were included in this review, and a meta-analysis of 16 outcomes was conducted.

According to the findings, there was evidence of low to very low quality that HM significantly improved FEV1 (L), FEV1 (%) and 6MWD, as well as evidence of moderate quality that HM significantly improved FVC (L) compared to placebo. However, according to evidence of low quality, there was no significant difference in FEV1/FVC (%) or VC (L) between the groups. Interestingly, in subgroup analysis according to COPD status and treatment duration, significant improvements in the use of HM in AECOPD patients were observed when the treatment duration was ≤ 4 weeks, but not for stable COPD when the treatment duration was ≥2 months. HM significantly reduced the severity of dyspnea evaluated by mMRC in AECOPD patients (high quality), but not in stable COPD patients (low quality), compared to placebo. The HM group showed significantly better TER (low quality; high quality) and CAT (low quality; high quality) than the placebo group in both stable COPD and AECOPD, regardless of the treatment duration. In addition, HM significantly lowered the frequency of acute exacerbations in patients with stable COPD compared to placebo (moderate quality). HM showed significant improvement in SGRQ compared to placebo in the total sample (low quality), but no significant improvement in stable COPD (very low quality) or AECOPD (moderate quality) in the subgroup analysis. Regarding quality of life, HM significantly improved the psychological domain evaluated by WHOQOL-BREF compared to placebo in AECOPD (high quality), but the opposite was seen in terms of social relationships (high quality). Regarding adverse events, no significant differences were observed between the HM and placebo groups, either overall or in any subgroup (low to high quality).

Overall, the methodological quality of the included RCTs was not the best, and in particular, the description of allocation concealment and assessor blinding was inadequate or insufficient. Although this review only included RCTs using placebo, which was thought to be indistinguishable from active HM, there is still a risk of selection bias or detection bias, suggesting that skewed conclusions may be drawn from actual results. In addition, most of the results of the quality of evidence evaluated as GRADE were low to moderate, and the evidence of high quality was insufficient. Therefore, the findings of this review are likely to be significantly affected by the subsequent implementation of larger RCTs with higher methodological quality. At the same time, however, the findings of this review show that low or moderate levels of evidence revealed that HM is likely to improve respiratory function in COPD patients, especially AECOPD patients; reduce acute exacerbation in patients with stable COPD; and improve COPD symptoms compared to placebo.

### Clinical Implications

Research shows that many COPD patients prefer complementary and alternative medicine (CAM) modalities (Abadoglu et al., 2008; Şahin and Şahin 2013). HM is a representative CAM modality, but in terms of evidence-based medicine, high quality evidence is needed to draw conclusions about its use for COPD (Chen et al., 2014). The findings in this review show that HM has the potential to help improve some respiratory functions, COPD symptoms, and some aspects of quality of life in COPD patients compared to placebo. Moreover, although not our outcome of interest, some RCTs included provided biological outcomes suggesting the anti-inflammatory effects of HM in COPD patients. This is consistent with recent findings suggesting the underlying mechanism of HM in COPD (Santana et al., 2016; Kim et al., 2017; Yang et al., 2020). In addition, flavonoids, alkaloids, and terpenoids, which are major components of several HMs, have the potential to positively affect COPD through various mechanisms, such as lessening of inflammation and oxidative stress, inhibition of cellular senescence, restoration of corticosteroid sensitivity, and improvement of pulmonary histology as well as pulmonary function (Santana et al., 2016; Yang et al., 2020). The possible underlying therapeutic mechanisms of HM shared with conventional medicine for COPD may raise expectations for a positive synergistic effect in treating COPD, but, conversely, may raise concerns about herb-drug interactions (Pao et al., 2012). Although our review suggests that HM has the potential to improve some outcomes of COPD patients prior to placebo, in clinical settings, it may not only have synergistic effects with conventional medicine for COPD, but may also have potentially negative herb-drug interaction which should be further studied before making recommendations regarding the use of HM in COPD patients. Moreover, the findings in this review suggest that HM may improve some aspects of respiratory function, especially in AECOPD patients. Given that conventional medicine could be a major treatment strategy in AECOPD patients, studies regarding herb-drug interactions could further encourage or hinder the use of HM in this population. However, the effect of HM in reducing acute exacerbation in stable COPD patients seem to be steadily garnering much research attention.

### Strengths and Limitations

The strength of this systematic review is that it was comprehensive and was conducted without limiting the participants’ COPD status. It was also a critical review conducted only on studies using placebo interventions that could be recognized as meaningful. However, due to the following limitations, the findings of this review should be interpreted with caution:

**First**, the methodological quality of the included studies was not the best. Although this review included only RCTs with placebo HM, in which the difference in taste, aroma, and texture from active HM cannot be discerned, most included RCTs were still not free from the risk of selection bias and detection bias. **Second**, all included studies were conducted in China. Considering that HM based on EATM theory is mostly implemented in East Asian countries such as China, Japan, Korea, and Taiwan, this is not a strange result, but regionally uneven publication hinders generalization of the results. In addition, visual asymmetry was observed in the funnel plot of the two outcomes, suggesting potential publication bias. This suggests that a pre-registered, larger, more rigorous RCT is needed for this topic in the future, which ideally should be evenly distributed regionally, ethnically, and racially. **Third**, the HMs used in the included studies were heterogeneous, suggesting the necessity of developing and using standardized HM. In recent years, for example, standardized HMs such as PM014 have been developed, and further clinical research is expected (Jung et al., 2013). Moreover, some frequently used herbs for treating COPD, including *Poria cocos (Schw.) Wolf [*Polyporaceae*; Poria(Hoelen)*] and *Citrus unshiu Markovich [*Rutaceae*; Citri unshius pericarpium]* found in this review, can be considered in the development of standardized HMs, such as PM014. Moreover, some herbs that were frequently used in studies on stable and unclear COPD including *Codonopsis pilosulae (Fr.) Nannf [*Campanulaceae*; Codonopsis pilosulae radix]*, *Atractylodes macrocepha-la Koidz [*Asteraceae*; Atractylodis rhizoma alba]*, *Cornus officinalis Sieb. et Zucc [*Cornaceae*; Corni fructus]*, *Rehmannia glutinosa (Gaertner) Libosch [*Scrophulariaceae*; Rehmanniae radix preparat]*, and *Astragalus membranaceus Bunge [*Leguminosae*; Astragali radix]* were not used in studies on AECOPD. *Codonopsis pilosulae (Fr.) Nannf. [*Campanulaceae*; Codonopsis pilosulae radix]* was the most frequently used herb only in studies on stable COPD. In contrast, *Carthamus tinctorius L. [*Asteraceae*; Carthami flos]*, *Pheretima aspergillum (E. Perrier) [*Lumbricidae*; Lumbricus corpus]*, *Sinapis alba L [*Brassicaceae*; Sinapis semen]*, and *Raphanus sativus L. [*Brassicaceae*; Raphani semen]* were the only herbs used in studies on AECOPD. The differential use of these herbs may be considered clinically for COPD and the development of standardized HMs. Further, small number of studies were included and the use of HM was heterogeneous; therefore, it was difficult to analyze the effect of each of these herbs on the individual symptoms of COPD. The heterogeneity of the HM may be due to the broad intervention criteria established in this review. Future research may try to further analyze focused on frequently used HMs, HMs containing specific important herbs, or standardized HMs. It is expected that the findings of this review can be used as a reference in this further research. **Fourth**, among the included studies, most have specified the status of COPD, such as stable COPD or AECOPD, but there were also studies that did not. However, these two conditions differ pathologically, and it is likely that some of the outcomes may show differences in the treatment responsiveness of HM according to our results. Thus, future studies should require a clear description of the participants’ COPD status. **Fifth**, we included studies describing the similarity between placebo and original HM with regard to taste or aroma as well as visual characteristics for only high-quality trials having a minimal relevant performance bias. However, studies might not fully explain the homogeneity of HM and placebo, resulting in the exclusion of a significant number of large-scale studies from the study selection process. Therefore, the inclusion of these studies may have affected the results of this study. **Sixth**, in the 14 trials, three of them used HMs with additional components; however, reports were not found on how they and the corresponding placebo were manufactured and blinded. Emails were sent to the authors to confirm this; however, no response was received. Therefore, caution is needed when interpreting the results of these studies. **Finally**, most of the included studies described the quality control of the used HM, but only a study by Wang et al. (2014) reported chemical analysis of HM. However, the included studies are HMs composed of multiherbs, and chemical analysis such as high performance liquid chromatography needs to be performed and reported to explore its potential mechanism. In future studies, it is highly recommended not only to standardize the composition of HM, but also to report the component analysis results.

## Conclusion

According to the results of this systematic review, very low to moderate evidence suggests that HM has the potential to help improve some COPD patients’ respiratory functions, especially those with AECOPD; COPD symptoms; and quality of life compared to placebo. However, these findings are challenged by the poor methodological quality of the included studies, the heterogeneity of HMs used, and potential publication bias. Therefore, the findings could be significantly influenced by further larger, more rigorous RCTs on this topic. Moreover, it may also be recommended to develop standardized HMs focused on some individual herbs that are frequently used or expected to play an important role in patients with COPD, and to elucidate the underlying mechanisms.

## Abbreviations

AECOPD, acute exacerbation of chronic obstructive pulmonary disease; CAT, chronic obstructive pulmonary disease assessment test; CI, confidence interval; COPD, chronic obstructive pulmonary disease; EATM, East Asian traditional medicine; FEV1, forced expiratory volume in 1 s; FVC, forced vital capacity; GRADE, grading of recommendations assessment development and evaluations; HM, herbal medicine; IL, interleukin; MD, mean difference; mMRC, the modified Medical Research Council; PRISMA, preferred reporting items for systematic reviews and meta-analyses; RCT, randomized controlled trial; RR, risk ratio; SGRQ, St. George respiratory questionnaire; TER, total effective rate; TGF, transforming growth factor; TNF, tumor necrosis factor; VC, vital capacity; WHOQOL-BREF, World Health Organization quality of life instruments-abbreviated version; 6MWD, 6-min walk distance.
