# Supplementary material for: Herbal Medicine Compared to Placebo for Chronic Obstructive Pulmonary Disease: A Systematic Review and Meta-Analysis
Source: Front Pharmacol. 2021 Oct 20;12:717570. doi: 10.3389/fphar.2021.717570 (PMC8564496; doi:10.3389/fphar.2021.717570)
Supplement: Supplementary file 1 [file Table1.DOCX]

Table 1. Baseline characteristics of the included studies

| **Study** | **Sample size**  **(included →analyzed)** | **Mean age  (yr)** | **Sex (Male:Female)** | **Population (diagnostic criteria)** | **(A) Treatment intervention** | **(B) Control intervention** | **Outcome** | **Adverse event** |
| --- | --- | --- | --- | --- | --- | --- | --- | --- |
| Li 2011 | 65(34:31)→65(34:31) | (A) 63.90 ± 11.37 (B) 65.70 ± 12.31 | (A) 23:11 (B) 19:12 | -AECOPD (GOLD, 2007 Guidelines of Respiratory Diseases Branch of the Chinese Medical Association) -Blood stasis | HM | Placebo | 1. TER (clinical symptom and sign) 2. absorption condition of chest lesions shown in the images 3. arterial blood gas analysis 4. FVC (L) 5. FEV1 (L) 6. FEV1 (%) 7. TER (TCM syndrome score) 8. WHOQOL-BREF 9. SGRQ 10. PFSDQ-MM | NR |
| Liu 2011 | 263(178:85)→216(149:67) | (A) 68.95 ± 8.66 (B) 68.38 ± 8.77 | (A) 158:20 (B) 70:15 | -stable COPD (GOLD) | HM | Placebo | 1. Frequency of acute exacerbation 2. FEV1 (L) 3. FEV1/FVC (%) 4. FEV1 (%) 5. nutritional statues 6. 6MWM 7. SGRQ 8. TCM syndrome score 9. TER (TCM syndrome score) 10. BODE index | (A) cold limbs (1), lip color turning red (1), urine sugar (1), diarrhea (1), proteinuria (1) (B) abdominal distension (1), stomachache (1), mouth ulcer (1) |
| Sun 2013 | 76(38:38)→70(36:34) | (A) 67.81 ± 8.61 (B) 67.26 ± 10.43 | (A) 17:19 (B) 17:17 | -stable COPD (2007 Guidelines of Respiratory Diseases Branch of the Chinese Medical Association) -Qi deficiency of spleen and kidney | HM | Placebo | 1. TCM syndrome score 2. TER (TCM syndrome score) 3. FEV1 (%) 4. CAT 5. inflammatory factor (IL-8, TNF-α) | none |
| Guo 2014 | 140(70:70)→130(69:61) | NR | NR | -stable COPD (GOLD) -Deficiency of lung, spleen, and kidney accompanied with retention of phlegm and blood stasis | HM | Placebo | 1. TER (TCM syndrome score) 2. Frequency of acute exacerbation 3. TCM syndrome score 4. mMRC 5. SGRQ 6. FEV1 (L) 7. FVC (L) 8. FEV1 (%) 9. FEV1/FVC (%) 10. MMEF 75/25 (%) 11. PEF (L/min) 12. inflammatory factor (IL-6, IL-8, TNF-alpha, TGF-beta1) | none |
| Liu 2014 | 244(122:122)→244(122:122) | (A) 70.1 ± 9.8 (B) 70.7 ± 9.8 | (A) 88:34 (B) 82:40 | -AECOPD (GOLD, 2007 Guidelines of Respiratory Diseases Branch of the Chinese Medical Association)  -Phlegm-heat obstructing the lungs | HM | Placebo | 1. Total symptom scores (cough, phlegm, wheezing, chest congestion) 2. FEV1 (L) 3. FVC (L) 4. FEV1 (%) 5. arterial blood gas analysis 6. pro-inflammatory biomarkers 7. oxidant/antioxidant balance | (A) mild diarrhea (6) (B) mild diarrhea (2) |
| Wang 2014 | 331 (109:109:113)→262(82:89:91) | (A1) 62.43 ± 9.04 (A2) 61.51 ± 8.79 (B) 62.68 ± 8.10 | (A1) 64:18 (A2) 62:27 (B) 69:22 | -stable COPD (GOLD) -Deficiency of qi, kidney qi, or kidney yang | HM | Placebo | 1. VC (L) 2. FEV1 (%) 3. FEV1/FVC (%) (pre-bronchodilator) 4. FEV1/FVC (%) (post-bronchodilator) 5. PEF (L/S) 6. Frequency of acute exacerbation 7. SGRQ  8. BODE index 9. 6MWD 10. WHOQOL-BREF (psychological field score) 11. inflammatory factor (IL-6, IL-8, IL-10, IL-17, IL-1β, TGF-β1, TNF-α) (unclear unit) 12. cortisol (HPA-axis function) | (A1) 13.54%  (A2) 14.14%  (B) 16.50%  *Raw data was not reported (no significant difference between groups). There were no serious adverse events reported. |
| Chen 2015 | 160(80:80)→160(80:80) | (A) 73.25 ± 11.56 (B) 72.45 ± 12.11 | (A) 62:18 (B) 60:20 | -COPD (2013 Guidelines of Respiratory Diseases Branch of the Chinese Medical Association) -Deficiency | HM | Placebo | 1. TER (clinical symptom and sign) 2. TCM syndrome score 3. FEV1 (L) 4. FEV1/FVC (%) 5. SGRQ 6. 6MWD | None |
| Shi 2016 | 63(32:31)→63(32:31) | (A) 57.88 ± 7.246 (B) 58.06 ± 6.527 | (A) 17:15 (B) 16:15 | -stable COPD (GOLD, 2013 Guidelines of Respiratory Diseases Branch of the Chinese Medical Association) -Qi deficiency of heart and lung | HM | Placebo | 1. TCM syndrome score 2. HAMA 3. CAT 4. TER (TCM syndrome score) 5. TER (HAMA) | (A) sweating (1)  (B) dry mouth (1), sweating (1), lethargy (1) |
| Gao 2017 | 40(20:20)→40(20:20) | (A) 60.34 ± 8.27 (B) 62.44 ± 10.09 | (A) 8:12 (B) 9:11 | -COPD (<Internal Medicine> 6th Edition) -Phlegm-heat obstructing the lungs | HM | Placebo | 1. CAT 2. TCM syndrome score 3. 6MWD 4. FEV1 (L) 5. FEV1/FVC (%) 6. TER (TCM syndrome score) | NR |
| Hong 2018 | 60(30:30)→55(28:27) | (A) 67.9 ± 7.8 (B) 68.6 ± 7.7 | (A) 28:0 (B) 26:1 | -stable COPD (2007 Guidelines of Respiratory Diseases Branch of the Chinese Medical Association) -Deficiency of lung, spleen, and kidney accompanied with retention of phlegm and blood stasis | HM | Placebo | 1. TCM syndrome score  2. FEV1 (%) 3. FEV1/FVC (%) | (A) sputum changed from thick white to light yellow (1) (B) gastric discomfort (2), diarrhea (1) |
| Huang 2019 | 80(40:40)→80(40:40) | (A) 65.6 ± 4.2 (B) 64.9 ± 4.9 | (A) 26:14 (B) 23:17 | -AECOPD (2013 Guidelines of Respiratory Diseases Branch of the Chinese Medical Association)  -Blood stasis | HM | Placebo | 1. TER (clinical symptom and sign) 2. FEV1 (L) 3. FVC (L) 4. FEV1 (%) 5. inflammatory factor (IL-17, IL-10, 8-iso-prostaglandin) | NR |
| Jin 2019 | 60(30:30)→54(28:26) | (A) 69.12 ± 4.86 (B) 68.54 ± 3.51 | (A) 18:10 (B) 14:12 | -stable COPD (2013 Guidelines of Respiratory Diseases Branch of the Chinese Medical Association)  -Qi deficiency of lung and spleen | HM | Placebo | 1. TER (frequency of acute exacerbation) 2. TCM syndrome score 3. FFMI 4. hand grip strength 5. TWEAK level | NR |
| Luo 2019 | 140(70:70)→140(70:70) | (A) 71.91 ± 3.51 (B) 71.88 ± 3.46 | (A) 46:24 (B) 45:25 | -AECOPD (2007 Guidelines of Respiratory Diseases Branch of the Chinese Medical Association) -Phlegm-turbidity obstructing the lungs | HM | Placebo | 1. IL-32 2. FEV1 (L) 3. FEV1 (%) 4. MMRC 5. CAT 6. 6MWD 7. TER (clinical symptom and sign) | NR |
| Hu 2020 | 60(30:30)→60(30:30) | (A) 72.7 ± 9.2 (B) 75.3 ± 7.6 | (A) 24:6 (B) 24:6 | -stable COPD (GOLD) | HM | Placebo | 1. Frequency of acute exacerbation (yr) 2. Frequency of separate acute exacerbation associated with striking air pollution (yr) 3. Number of deteriorated respiratory symptoms related to striking air pollution (yr) 4. CAT 5. mMRC | (A) diarrhea (2) (B) none |

**Abbreviations.** AECOPD, acute exacerbations of chronic obstructive pulmonary disease; BODE, body-mass index, obstruction of airways, dyspnea, exercise capacity; CAT, chronic obstructive pulmonary disease assessment test; COPD, chronic obstructive pulmonary disease; FEV1, forced expiratory volume in one second; FFMI, fat free mass index; FVC, forced vital capacity; GOLD, global initiative for chronic obstructive lung disease; HAMA, Hamilton anxiety rating scale; HM, herbal medicine; HPA, hypothalamic-pituitary-adrenal; IL, interleukin; MMEF, mean maximum expiratory flow; mMRC, modified medical research council dyspnea scale; NR, not recorded; PEF, peak expiratory flow; PFSDQ-MM, modified version of pulmonary functional status and dyspnea questionnaire; SGRQ, St. George respiratory questionnaire; TCM, traditional Chinese medicine; TER, total effective rate; TGF, transforming growth factor; TNF, tumor necrosis factor; TWEAK, tumor necrofacies-like weak inducer of apoptosis; VC, vital capacity; WHOQOL-BREF, World Health Organization quality of life instruments-abbreviated version; 6MWD, 6-minute walking distance.
