# Supplementary material for: Herbal Medicine Compared to Placebo for Chronic Obstructive Pulmonary Disease: A Systematic Review and Meta-Analysis
Source: Front Pharmacol. 2021 Oct 20;12:717570. doi: 10.3389/fphar.2021.717570 (PMC8564496; doi:10.3389/fphar.2021.717570)
Supplement: Supplementary file 2 [file Table2.docx]

Table 2. Detailed characteristics of herbal medicine used

| **Study** | **Herbal medicine** | **Dosage form** | **Basic component (for 1 day)** | **Additional component** | **Quality control reported / Chemical analysis reported** | **Administration period / follow-up** |  |
| --- | --- | --- | --- | --- | --- | --- | --- |
| **Stable COPD** | | | | | | | |
| Liu 2011 | Jianpi Yifei II recipe | granule | *Codonopsis pilosulae (Fr.) Nannf. [Campanulaceae; Codonopsis pilosulae radix], Astragalus membranaceus Bunge [Leguminosae; Astragali radix], Bupleurum falcatum Linne [Apiaceae; Bupleuri radix], Vitex rotundifolia L. Fil. [Verbenaceae; Viticis fructus]* et al. (Dosage not listed) | None | N / N | 2 months / 4 months |  |
| Sun 2013 | Sijunzi-tang + Jinkui Shenqi pills | decoction | *Panax ginseng C. A. Mey. [Araliaceae; Ginseng radix]* 10 g, *Poria cocos (Schw.) Wolf [Polyporaceae; Poria(Hoelen)]* 10 g, *Atractylodes macrocepha-la Koidz [Asteraceae; Atractylodis rhizoma alba]* 10 g, *Glycyrrhiza uralensis Fisch. [Leguminosae; Glycyrrhizae radix]* 6 g, *Cinnamomum cassia Blume [Lauraceae; Cinnamomi ramulus]* 10 g, *Rehmannia glutinosa (Gaertner) Libosch. [Scrophulariaceae; Rehmanniae radix preparat]* 10 g, *Cornus officinalis Sieb. et Zucc. [Cornaceae; Corni fructus]* 10 g, *Dioscorea batatas Decne. [Dioscoreaceae; Dioscoreae rhizoma]* 10 g, *Paeonia suffruticosa Andrews [Ranunculaceae; Moutan radicis cortex]* 10 g, *Alisma orientalis (Sam) Juzep [Alismataceae; Alismatis rhizoma]* 10 g, *Aconitum carmichaeli Debx [Ranunculaceae; Aconiti lateralis radix preparata]* 10 g | None | Y – produced by the Chinese Pharmacy of the Affiliated Hospital of Chengdu University of TCM / N | 4 weeks / none |  |
| Guo 2014 | Bufei granule | granule | *Codonopsis pilosulae (Fr.) Nannf. [Campanulaceae; Codonopsis pilosulae radix], Rehmannia glutinosa (Gaertner) Libosch. [Scrophulariaceae; Rehmanniae radix preparat], Cornus officinalis Sieb. et Zucc. [Cornaceae; Corni fructus], Ephedra sinica Stapf. [Ephedraceae; Ephedrae herba], Citrus unshiu Markovich [Rutaceae; Citri unshius pericarpium]* (Dosage not listed) | None | Y – produced by Pharmaceutical Centre of the Second Affiliated Hospital of Tianjin University of TCM (production batch number: 200702003) / N | 12 weeks / 12 months |  |
| Wang 2014 | (A1) Bushen Yiqi granule + placebo Bushen Fangchaun tablet (A2) Bushen Fangchuan tablet + placebo Bushen Yiqi granule | granule and tablet | (A1) *Astragalus membranaceus Bunge [Leguminosae; Astragali radix], Epimedium koreanum Nakai [Berberidaceae; Epimedii herba], Rehmannia glutinosa var. purpurea (Makino) Makino et Nemoto [Scrophulariaceae; Rehmanniae radix recens]* (Dosage not listed) (A2) *Epimedium koreanum Nakai [Berberidaceae; Epimedii herba], Rehmannia glutinosa var. purpurea (Makino) Makino et Nemoto [Scrophulariaceae; Rehmanniae radix recens], Aconitum carmichaeli Debx [Ranunculaceae; Aconiti lateralis radix preparata], Psoralea corylifolia L. [Leguminosae; Psoraleae fructus], Cuscuta chinensis Lam. [Convolvulaceae; Cuscutae semen], Rehmannia glutinosa (Gaertner) Libosch. [Scrophulariaceae; Rehmanniae radix preparat], Dioscorea batatas Decne. [Dioscoreaceae; Dioscoreae rhizoma], Citrus unshiu Markovich [Rutaceae; Citri unshius pericarpium]* (Dosage not listed) | None | Y – produced by the second TCM factory of Taiji Group of Sichuan province and Tianjiang Pharmacy company Ltd of Jiangsu province / Y – HPLC | 180 days / 180 days |  |
| Shi 2016 | No name | decoction | *Codonopsis pilosulae (Fr.) Nannf. [Campanulaceae; Codonopsis pilosulae radix]* 15 g, *Salvia miltiorrhiza Bunge [Lamiaceae; Salviae miltiorrhizae radix et rhizoma]* 15 g, *Adenophora triphylla var. japonica Hara [Campanulaceae; Adenophorae radix]* 30 g, *Pteria martensii (Dunker) [Pteridae; Margaritifera usta concha]* 30 g, *Poria cocos (Schw.) Wolf [Polyporaceae; Poria(Hoelen)]* 30 g, *Paeonia lactiflora Pallas [Paeoniaceae; Paeoniae radix]* 10 g, *Schizandra chinensis (Turcz.) Baill. [Magnoliaceae; Schisandrae fructus]* 6 g, *Poncirus trifoliata Rafin. [Rutaceae; Aurantii fructus immaturus]* 12 g | None | N / N | 14 days / none |  |
| Hong 2018 | Yufeining granule | granule | *Codonopsis pilosulae (Fr.) Nannf. [Campanulaceae; Codonopsis pilosulae radix], Astragalus membranaceus Bunge [Leguminosae; Astragali radix], Saposhnikovia divaricata Schiskin [Apiaceae; Saposhnikoviae radix], Atractylodes macrocepha-la Koidz [Asteraceae; Atractylodis rhizoma alba], Juglans sinensis Dode [Juglandaceae; Semen juglandis], Cuscuta chinensis Lam. [Convolvulaceae; Cuscutae semen], Cornus officinalis Sieb. et Zucc. [Cornaceae; Corni fructus], Schizandra chinensis (Turcz.) Baill. [Magnoliaceae; Schisandrae fructus], Prunus armeniaca L. var. ansu Maxim. [Rosaceae; Armeniacae semen], Fritillaria thunbergii Miq. [Liliaceae; Fritillariae thunbergii bulbus], Trichosanthes kirilowii Maxim. [Cucurbitaceae; Trichosanthis fructus], Lepidium apetalum Willd. [Brassicaceae; Lepidii seu descurainiae semen], Prunus persica (L.) Batsch [Rosaceae; Persicae semen], Salvia miltiorrhiza Bunge [Lamiaceae; Salviae miltiorrhizae radix et rhizoma]* et al. (Dosage not listed) | None | Y – provided by Zhangzhou traditional Chinese medicine Hospital Affiliated to Fujian University of TCM / N | 8 weeks / none |  |
| Jin 2019 | Yiqi Gubiao pills | pill | *Codonopsis pilosulae (Fr.) Nannf. [Campanulaceae; Codonopsis pilosulae radix], Atractylodes macrocepha-la Koidz [Asteraceae; Atractylodis rhizoma alba], Poria cocos (Schw.) Wolf [Polyporaceae; Poria(Hoelen)], Citrus unshiu Markovich [Rutaceae; Citri unshius pericarpium], Pinellia ternata (Thunb.) Breit. [Araceae; Pinelliae rhizoma], Coix lachryma-jobi var. ma-yeun (Roman.) Stapf [Gramineae; Coicis semen], Triticum aestivum L. [Gramineae; Tritici fructus levis], Perilla frutescens var. acuta Kudo [Labiatae; Perilliae semen], Tussilago farfara L. [Asteraceae; Farfarae flos], Scutellaria baicalensis Georgi [Labiatae; Scutellariae radix], Fritillaria thunbergii Miq. [Liliaceae; Fritillariae thunbergii bulbus], Eriobotrya japonica Lindl. [Rosaceae; Eriobotryae folium], Saposhnikovia divaricata Schiskin [Apiaceae; Saposhnikoviae radix]* (Dosage not listed) | None | Y – produced by Xinjiang Pharmaceutical Factory, provided by the Central Pharmacy of the Affiliated Hospital of TCM of Xinjiang Medical University (production batch number: 20170431) / N | 12 weeks / none |  |
| Hu 2020 | Qingfei Yihuo capsule | capsule | *Scutellaria baicalensis Georgi [Labiatae; Scutellariae radix], Gardenia jasminoides var. grandiflora (Lour.) Nakai [Rubiaceae; Gardeniae fructus], Trichosanthes kirilowii var. japonica Kitamura [Cucurbitaceae; Trichosanthis radix], Platycodon grandiflorum (Jacq.) A. DC. [Campanulaceae; Platycodi radix], Anemarrhena asphodeloides Bunge [Haemodoraceae; Anemarrhenae rhizoma], Rheum palmatum L. [Polygonaceae; Rhei radix et rhizoma], Peucedanum decursivum (Miq.) Maxim. [Apiaceae; Peucedani radix], Phellodendron amurense Rupr. [Rutaceae; Phellodendri cortex], Sophora flavescens Ait. [Leguminosae; Sophorae radix]* (Dosage not listed) | None | Y – produced by Shanghai Fangxin Health and Science Development Ltd., China and met the drug quality standards (production batch number: 040601) / N | 15 days / 1 year |  |
| **AECOPD** | | | | | | | |
| Li 2011 | Huoxue Huayu recipe | granule | *Cnidium officinale Makino [Apiaceae; Cnidii rhizoma], Paeonia lactiflora Pallas [Paeoniaceae; Paeoniae radix], Prunus persica (L.) Batsch [Rosaceae; Persicae semen], Carthamus tinctorius L. [Asteraceae; Carthami flos]* et al. (Dosage not listed) | -phlegm-heat/phlegm-dampness: clear and resolve heat-phlegm (*Trichosanthes kirilowii Maxim. [Cucurbitaceae; Trichosanthis fructus], Pinellia ternata (Thunb.) Breit. [Araceae; Pinelliae rhizoma], Fritillaria cirrhosa D. Don. [Liliaceae; Fritillariae cirrhosae bulbus], Gardenia jasminoides var. grandiflora (Lour.) Nakai [Rubiaceae; Gardeniae fructus], Morus alba L. [Moraceae; Mori radicis cortex], Scutellaria baicalensis Georgi [Labiatae; Scutellariae radix]*), dry dampness to resolve phlegm (*Pinellia ternata (Thunb.) Breit. [Araceae; Pinelliae rhizoma], Magnolia officinalis Rehder et Wilson [Magnoliaceae; Magnoliae cortex], Citrus unshiu Markovich [Rutaceae; Citri unshius pericarpium], Allium macrostemon Bge. [Liliaceae; Allii macrostemi bulbus], Poria cocos (Schw.) Wolf [Polyporaceae; Poria(Hoelen)], Poncirus trifoliata Rafin. [Rutaceae; Aurantii fructus immaturus]* et al.) (Dosage not listed) | Y – provided by Jiangyin Tianjiang Pharmaceutical Co., Ltd. / N | 14 days / none |  |
| Liu 2014 | Xuanbai Chengqi granule | granule | *Gypsum [Gypsum fibrosum], Rheum palmatum L. [Polygonaceae; Rhei radix et rhizoma], Prunus armeniaca L. var. ansu Maxim. [Rosaceae; Armeniacae semen], Trichosanthes kirilowii Maxim. [Cucurbitaceae; Trichosanthis cortex]* (Dosage not listed) | None | Y – produced by Jiang Yin Tian Jiang Pharmaceutical Co. Ltd. Under good manufacturing practice regulations of China (Approval number: 0905301-4) / N | 10 days / none |  |
| Huang 2019 | Mengshiguntan-wan | decoction | *Chlorite-schist [Chalcocitum]* 30 g, *Rheum palmatum L. [Polygonaceae; Rhei radix et rhizoma]* 6 g, *Scutellaria baicalensis Georgi [Labiatae; Scutellariae radix]* 10 g, *Pinellia ternata (Thunb.) Breit. [Araceae; Pinelliae rhizoma]* 10 g, *Fritillaria thunbergii Miq. [Liliaceae; Fritillariae thunbergii bulbus]* 10 g, *Gardenia jasminoides var. grandiflora (Lour.) Nakai [Rubiaceae; Gardeniae fructus]* 10 g, *Morus alba L. [Moraceae; Mori radicis cortex]* 10 g, *Prunus armeniaca L. var. ansu Maxim. [Rosaceae; Armeniacae semen]* 10 g, *Poria cocos (Schw.) Wolf [Polyporaceae; Poria(Hoelen)]* 10 g, *Citrus unshiu Markovich [Rutaceae; Citri unshius pericarpium]* 10 g, *Citrus unshiu Markovich [Rutaceae; Citri unshius immaturi pericarpium]* 10 g, *Prunus persica (L.) Batsch [Rosaceae; Persicae semen]* 15 g, *Paeonia lactiflora Pallas [Paeoniaceae; Paeoniae radix]* 15 g, *Pheretima aspergillum (E. Perrier) [Lumbricidae; Lumbricus corpus]* 15 g, *Liriope platyphylla Wang et Tang [Liliaceae; Liriopes radix]* 15 g, *Glycyrrhiza uralensis Fisch. [Leguminosae; Glycyrrhizae radix]* 6 g | None | Y – herbs were purchased from the Chinese pharmacy of the Affiliated Hospital of Jiangxi University of TCM. And automatic airtight decocting machine (Beijing Donghuayuan Medical Equipment Co., Ltd.) was used to make decoction. / N | 2 weeks / none |  |
| Luo 2019 | modified Sanzi Yangqin decoction | decoction | *Perilla frutescens var. acuta Kudo [Labiatae; Perilliae semen]* 10 g, *Sinapis alba L. [Brassicaceae; Sinapis semen]* 10 g, *Raphanus sativus L. [Brassicaceae; Raphani semen]* 10 g, *Pinellia ternata (Thunb.) Breit. [Araceae; Pinelliae rhizoma]* 10 g, *Poria cocos (Schw.) Wolf [Polyporaceae; Poria(Hoelen)]* 10 g, *Citrus unshiu Markovich [Rutaceae; Citri unshius pericarpium]* 6 g, *Glycyrrhiza uralensis Fisch. [Leguminosae; Glycyrrhizae radix]* 5 g | None | Y – provided by the Department of Pharmacy, Shunde District Hospital of TCM, Foshan City / N | 14 days / none |  |
| **Unclear COPD** | | | | | | | |
| Chen 2015 | Lifei Yishen recipe | decoction | *Rehmannia glutinosa (Gaertner) Libosch. [Scrophulariaceae; Rehmanniae radix preparat]* 15 g, *Cornus officinalis Sieb. et Zucc. [Cornaceae; Corni fructus]* 15 g, *Schizandra chinensis (Turcz.) Baill. [Magnoliaceae; Schisandrae fructus]* 5 g, *Poria cocos (Schw.) Wolf [Polyporaceae; Poria(Hoelen)]* 15 g, *Aconitum carmichaeli Debx [Ranunculaceae; Aconiti lateralis radix preparata]* 6 g, *Cinnamomum cassia Blume [Lauraceae; Cinnamomi cortex spissus]* 6 g, *Aquilaria agallocha Roxb. [Thymelaceae; Aquilariae resinatum lignum]* 10 g, *Astragalus membranaceus Bunge [Leguminosae; Astragali radix]* 15 g, *Liriope platyphylla Wang et Tang [Liliaceae; Liriopes radix]* 10 g, *Atractylodes macrocepha-la Koidz [Asteraceae; Atractylodis rhizoma alba]* 10 g, *Citrus unshiu Markovich [Rutaceae; Citri unshius pericarpium]* 10 g, *Glycyrrhiza uralensis Fisch. [Leguminosae; Glycyrrhizae radix]* 6 g | -phlegm: *Platycodon grandiflorum (Jacq.) A. DC. [Campanulaceae; Platycodi radix]* 15 g, *Perilla frutescens var. acuta Kudo [Labiatae; Perilliae semen]* 10 g -anorexia: Jiaosanxian 10 g (including *Hordeum vulgare L. [Gramineae; Hordei fructus germiniatus]*, *Crataegus pinnatifida Bge [Rosaceae; Crataegii fructus]*, and *Triticum aestivum L. [Gramineae; Massa medicata fermentata]*) -spleen qi deficiency: *Codonopsis pilosulae (Fr.) Nannf. [Campanulaceae; Codonopsis pilosulae radix]* 15 g | N / N | 4 months / none |  |
| Gao 2017 | Dingchuan-tang | decoction | *Ephedra sinica Stapf. [Ephedraceae; Ephedrae herba]* 10 g, *Ginkgo biloba L. [Ginkgoaceae; Ginkgonis semen]* 10 g, *Tussilago farfara L. [Asteraceae; Farfarae flos]* 15 g, *Pinellia ternata (Thunb.) Breit. [Araceae; Pinelliae rhizoma]* 15 g, *Perilla frutescens var. acuta Kudo [Labiatae; Perilliae semen]* 20 g, *Morus alba L. [Moraceae; Mori radicis cortex]* 10 g, *Scutellaria baicalensis Georgi [Labiatae; Scutellariae radix]* 20 g, *Prunus armeniaca L. var. ansu Maxim. [Rosaceae; Armeniacae semen]* 15 g, *Glycyrrhiza uralensis Fisch. [Leguminosae; Glycyrrhizae radix]* 5 g | -severe fever: *Eriobotrya japonica Lindl. [Rosaceae; Eriobotryae folium]* 10 g, *Gypsum [Gypsum fibrosum]* 10 g -spleen deficiency: *Poria cocos (Schw.) Wolf [Polyporaceae; Poria(Hoelen)]* 10 g, *Atractylodes macrocepha-la Koidz [Asteraceae; Atractylodis rhizoma alba]* 15 g, *Citrus unshiu Markovich [Rutaceae; Citri unshius pericarpium]* 10 g -blood stasis: *Panax noto-ginseng (Burk) f. H. Chen [Araliaceae; Notoginseng radix]* 10 g | Y – prepared by the Chinese pharmacy of Leshan TCM Hospital / N | 4 weeks / none |  |

**Abbreviation**. COPD, chronic obstructive pulmonary disease; HPC, high performance liquid chromatography; TCM, traditional Chinese medicine; N, no; Y, yes
