# Supplementary material for: Herbal Medicine Compared to Placebo for Chronic Obstructive Pulmonary Disease: A Systematic Review and Meta-Analysis
Source: Front Pharmacol. 2021 Oct 20;12:717570. doi: 10.3389/fphar.2021.717570 (PMC8564496; doi:10.3389/fphar.2021.717570)
Supplement: Supplementary file 3 [file Table5.DOCX]

**Appendix 2**. Search terms used in each database

**MEDLINE via PubMed**

|  | Searches | Results |
| --- | --- | --- |
| #1 | “Lung Diseases, Obstructive”[MH] OR (obstruct*[TIAB] AND (pulmonary[TIAB] OR airway*[TIAB] OR lung*[TIAB] OR airflow*[TIAB] OR bronch*[TIAB] OR respirat*[TIAB])) OR emphysema[TIAB] OR COPD[TIAB] OR COAD[TIAB] OR COBD[TIAB] OR AECB[TIAB] | 301,719 |
| #2 | “Plants, Medicinal”[MH] OR “Drugs, Chinese Herbal”[MH] OR “Medicine, Chinese Traditional”[MH] OR “Medicine, Kampo”[MH] OR “Medicine, Korean Traditional”[MH] OR “Herbal Medicine”[MH] OR “Prescription Drugs”[MH] OR “traditional Korean medicine”[TIAB] OR “traditional Chinese medicine”[TIAB] OR “traditional oriental medicine”[TIAB] OR “Kampo medicine”[TIAB] OR herb*[TIAB] OR decoction*[TIAB] OR botanic*[TIAB] | 228,442 |
| #3 | “Randomized Controlled Trial”[PT] OR “Controlled Clinical Trial”[PT] OR randomized[TIAB] OR placebo[TIAB] OR “Clinical Trials as Topic”[Mesh: noexp] OR randomly[TIAB] OR trial[TI] | 1,433,018 |
| #4 | animals[MH] NOT humans[MH] | 4,788,192 |
| #5 | #1 AND #2 AND #3 NOT #4 | **415** |

**EMBASE via Elsevier**

|  | Searches | Results |
| --- | --- | --- |
| #1 | ‘chronic obstructive lung disease’/exp OR ‘chronic obstructive lung disease’:ab,ti OR (obstruct*:ab,ti AND (pulmonary:ab,ti OR airway*:ab,ti OR lung*:ab,ti OR airflow*:ab,ti OR bronch*:ab,ti OR respirat*:ab,ti)) OR emphysema/exp OR emphysema:ab,ti OR COPD:ab,ti OR COAD:ab,ti OR COBD:ab,ti OR AECB:ab,ti | 302,585 |
| #2 | ‘medicinal plant’/exp OR ‘medicinal plant’:ab,ti OR ‘herbaceous agent’/exp OR ‘herbaceous agent’:ab,ti OR ‘chinese medicine’/exp OR ‘chinese medicine’:ab,ti OR ‘kampo medicine’/exp OR ‘kampo medicine’:ab,ti OR ‘kampo medicine (drug)’/exp OR ‘kampo medicine (drug)’:ab,ti OR ‘korean medicine’/exp OR ‘korean medicine’:ab,ti OR ‘herbal medicine’/exp OR ‘herbal medicine’:ab,ti OR ‘prescription drug’/exp OR ‘prescription drug’:ab,ti OR ‘oriental medicine’/exp OR ‘oriental medicine’:ab,ti OR ‘herb’/exp OR ‘herb’:ab,ti OR ‘decoction’:ab,ti OR ‘botanic’:ab,ti | 390,246 |
| #3 | 'crossover procedure':de OR 'double-blind procedure':de OR 'randomized controlled trial':de OR 'single-blind procedure':de OR (random* OR factorial* OR crossover* OR cross NEXT/1 over* OR placebo* OR doubl* NEAR/1 blind* OR singl* NEAR/1 blind* OR assign* OR allocat* OR volunteer*):de,ab,ti | 2,698,374 |
| #4 | #1 AND #2 AND #3 | **436** |

**CENTRAL**

|  | Searches | Results |
| --- | --- | --- |
| #1 | MeSH descriptor: [Lung Diseases, Obstructive] explode all trees | 19,769 |
| #2 | ((obstruct* AND (pulmonary OR airway* OR lung* OR airflow* OR bronch* OR respirat*)) OR emphysema OR COPD OR COAD OR COBD OR AECB):ti,ab,kw | 30,528 |
| #3 | MeSH descriptor: [Plants, Medicinal] explode all trees | 941 |
| #4 | MeSH descriptor: [Drugs, Chinese Herbal] explode all trees | 3,586 |
| #5 | MeSH descriptor: [Medicine, Chinese Traditional] explode all trees | 1,190 |
| #6 | MeSH descriptor: [Medicine, Kampo] explode all trees | 46 |
| #7 | MeSH descriptor: [Medicine, Korean Traditional] explode all trees | 33 |
| #8 | MeSH descriptor: [Herbal Medicine] explode all trees | 62 |
| #9 | MeSH descriptor: [Prescription Drugs] explode all trees | 107 |
| #10 | (“traditional Korean medicine” OR “traditional Chinese medicine” OR “traditional oriental medicine” OR “Kampo medicine” OR herb* OR decoction* OR botanic*):ti,ab,kw | 18,346 |
| #11 | ((#1 OR #2) AND (#3 OR #4 OR #5 OR #6 OR #7 OR #8 OR #9 OR #10)) in Trials | **438** |

**AMED via EBSCO**

|  | Searches | Results |
| --- | --- | --- |
| #1 | “Lung Diseases, Obstructive”[SU] OR (obstruct*[TX] AND (pulmonary[TX] OR airway*[TX] OR lung*[TX] OR airflow*[TX] OR bronch*[TX] OR respirat*[TX])) OR emphysema[TX] OR COPD[TX] OR COAD[TX] OR COBD[TX] OR AECB[TX] | 2,536 |
| #2 | “Plants, Medicinal”[SU] OR “Drugs, Chinese Herbal”[SU] OR “Medicine, Chinese Traditional”[SU] OR “Medicine, Kampo”[SU] OR “Medicine, Korean Traditional”[SU] OR “Herbal Medicine”[SU] OR “Prescription Drugs”[SU] OR “traditional Korean medicine”[TX] OR “traditional Chinese medicine”[TX] OR “traditional oriental medicine”[TX] OR “Kampo medicine”[TX] OR herb*[TX] OR decoction*[TX] OR botanic*[TX] | 34,541 |
| #3 | #1 AND #2 | **83** |

**KISS**

|  | Searches | Results |
| --- | --- | --- |
| #1 | (COPD OR 만성 폐쇄성 폐질환) AND 한약 | **0** |

**KCI**

|  | Searches | Results |
| --- | --- | --- |
| #1 | (COPD OR 만성 폐쇄성 폐질환) AND 한약 | **0** |

**CNKI**

|  | Searches | Results |
| --- | --- | --- |
| #1 | (SU='COPD'+'慢性阻塞性肺疾病'+'慢性阻塞性肺病'+'慢性阻塞性肺'+'肺气肿'+'慢性支气管炎'+'慢性气管炎'+'慢阻肺疾病'+'慢阻肺病'+'慢阻肺') AND (SU='中药'+'中医药'+'本草'+'汤'+'丸'+'散'+'颗粒'+'胶囊') AND (SU='安慰剂') | **79** |

**Wanfang data**

|  | Searches | Results |
| --- | --- | --- |
| #1 | (主题:COPD or 主题:慢性阻塞性肺疾病 or 主题:慢性阻塞性肺病 or 主题:慢性阻塞性肺 or 主题:肺气肿 or 主题:慢性支气管炎 or 主题:慢性气管炎 or 主题:慢阻肺疾病 or 主题:慢阻肺病 or 主题:慢阻肺) AND (主题:中药 or 主题:中医药 or 主题:本草 or 主题:汤 or 主题:丸 or 主题:散 or 主题:颗粒 or 主题:胶囊) AND (主题:安慰剂) | **188** |

**VIP.**

|  | Searches | Results |
| --- | --- | --- |
| #1 | (M=(COPD OR 慢性阻塞性肺疾病 OR 慢性阻塞性肺病 OR 慢性阻塞性肺 OR 肺气肿 OR 慢性支气管炎 OR 慢性气管炎 OR 慢阻肺疾病 OR 慢阻肺病 OR 慢阻肺) AND M=(中药 OR 中医药 OR 本草 OR 汤 OR 丸 OR 散 OR 颗粒 OR 胶囊) AND M=(安慰剂)) | **4** |
