# Supplementary material for: Herbal Medicine Compared to Placebo for Chronic Obstructive Pulmonary Disease: A Systematic Review and Meta-Analysis
Source: Front Pharmacol. 2021 Oct 20;12:717570. doi: 10.3389/fphar.2021.717570 (PMC8564496; doi:10.3389/fphar.2021.717570)
Supplement: Supplementary file 4 [file Table3.docx]

Table 3. Results of meta-analysis and quality of evidence

| **Outcomes** | **Subgroup** | **No. participants (RCTs)** | **Anticipated absolute effects (95% CI)** | | **Relative effect (95% CI)** | ***I^2^* value** | **Quality of evidence (GRADE)** | **Comments** |
| --- | --- | --- | --- | --- | --- | --- | --- | --- |
|  |  |  | **Risk with placebo group** | **Risk with herbal medicine group** |  |  |  |  |
| FEV1 (L) | Total | 1,041 (8) | - | MD 0.14 higher (0.03 to 0.24 higher) | - | 79 | ⨁⨁◯◯ LOW | Risk of bias (-1)^a^ Inconsistency (-1)^b^ |
| Subgroup 1 (COPD status) | Stable | 312 (2) | - | MD 0.16 higher (0.3 lower to 0.62 higher) | - | 94 | ⨁◯◯◯ VERY LOW | Risk of bias (-1)^a^ Inconsistency (-1)^b^ Imprecision (-1)^c^ |
|  | AECOPD | 529 (4) | - | MD 0.2 higher (0.08 to 0.32 higher) | - | 58 | ⨁⨁⨁◯ MODERATE | Inconsistency (-1)^b^ |
|  | Unclear | 200 (2) | - | MD 0.04 higher (0.03 lower to 0.11 higher) | - | 0 | ⨁⨁⨁◯ MODERATE | Imprecision (-1)^c^ |
| Subgroup 2 (Treatment duration) | ≤4 weeks | 569 (5) | - | MD 0.16 higher  (0.04 to 0.28 higher) | - | 69 | ⨁⨁⨁⨁ HIGH | - |
|  | ≥2 months | 472 (3) | - | MD 0.11 higher  (0.1 lower to 0.33 higher) | - | 87 | ⨁◯◯◯ VERY LOW | Risk of bias (-1)^a^ Inconsistency (-1)^b^ Imprecision (-1)^c^ |
| FEV1 (%) | Total | 1,228 (10) | - | MD 4.46 higher (1.93 to 6.99 higher) | - | 54 | ⨁◯◯◯ VERY LOW | Risk of bias (-1)^a^ Inconsistency (-1)^b^ Publication bias (-1)^d^ |
| Subgroup 1 (COPD status) | Stable | 699 (6) | - | MD 2.37 higher (1.84 lower to 6.58 higher) | - | 62 | ⨁◯◯◯ VERY LOW | Risk of bias (-1)^a^ Inconsistency (-1)^b^ Imprecision (-1)^c^ |
|  | AECOPD | 529 (4) | - | MD 6.57 higher (4.39 to 8.76 higher) | - | 0 | ⨁⨁⨁⨁ HIGH | - |
| Subgroup 2 (Treatment duration) | ≤4 weeks | 599 (5) | - | MD 6.47 higher  (4.35 to 8.59 higher) | - | 0 | ⨁⨁⨁⨁ HIGH | - |
|  | ≥2 months | 629 (5) | - | MD 2 higher  (2.82 lower to 6.82 higher) | - | 69 | ⨁◯◯◯ VERY LOW | Risk of bias (-1)^a^ Inconsistency (-1)^b^ Imprecision (-1)^c^ |
| FVC (L) | Total | 519 (4) | - | MD 0.22 higher (0.12 to 0.31 higher) | - | 16 | ⨁⨁⨁◯ MODERATE | Risk of bias (-1)^a^ |
| Subgroup (COPD status/Treatment duration) | Stable/≥2 months | 130 (1) | - | MD 0.4 higher (0.12 to 0.68 higher) | - | NA | ⨁⨁⨁◯ MODERATE | Risk of bias (-1)^a^ |
|  | AECOPD/≤4 weeks | 389 (3) | - | MD 0.19 higher (0.1 to 0.29 higher) | - | 0 | ⨁⨁⨁⨁ HIGH | - |
| FEV1/FVC (%) | Total | 829 (7) | - | MD 1.91 higher (0.72 lower to 4.55 higher) | - | 58 | ⨁⨁◯◯ LOW | Risk of bias (-1)^a^ Inconsistency (-1)^b^ |
| Subgroup 1 (COPD status) | Stable | 629 (5) | - | MD 2.26 higher (1.97 lower to 6.5 higher) | - | 70 | ⨁⨁◯◯ LOW | Risk of bias (-1)^a^ Inconsistency (-1)^b^ |
|  | Unclear | 200 (2) | - | MD 0.96 higher (0.88 lower to 2.79 higher) | - | 0 | ⨁⨁⨁⨁ HIGH | - |
| Subgroup 2 (Treatment duration) | ≤4 weeks | 40 (1) | - | MD 2 higher  (4.32 lower to 8.32 higher) |  | NA | ⨁⨁⨁◯ MODERATE | Imprecision (-1)^c^ |
|  | ≥2 months | 789 (6) | - | MD 1.92 higher  (1.06 lower to 4.9 higher) |  | 65 | ⨁◯◯◯ VERY LOW | Risk of bias (-1)^a^ Inconsistency (-1)^b^ Imprecision (-1)^c^ |
| VC (L) | Total (stable/≤4 weeks) | 262 (2) | - | MD 0 (0.18 lower to 0.18 higher) | - | 0 | ⨁⨁◯◯ LOW | Risk of bias (-1)^a^ Imprecision (-1)^c^ |
| 6MWD | Total | 818 (6) | - | MD 35.82 higher (15.26 to 56.38 higher) | - | 78 | ⨁⨁◯◯ LOW | Risk of bias (-1)^a^ Inconsistency (-1)^b^ |
| Subgroup 1 (COPD status) | Stable | 478 (3) | - | MD 12.87 higher (14.88 lower to 40.62 higher) | - | 48 | ⨁⨁◯◯ LOW | Risk of bias (-1)^a^ Imprecision (-1)^c^ |
|  | AECOPD | 140 (1) | - | MD 48.02 higher (42.74 to 53.3 higher) | - | NA | ⨁⨁⨁⨁ HIGH | - |
|  | Unclear | 200 (2) | - | MD 57.84 higher (1.47 to 114.2 higher) | - | 88 | ⨁⨁⨁◯ MODERATE | Inconsistency (-1)^b^ |
| Subgroup 2 (Treatment duration) | ≤4 weeks | 180 (2) | - | MD 43.08 higher  (27.01 to 59.15 higher) | - | 49 | ⨁⨁⨁⨁ HIGH | - |
|  | ≥2 months | 638 (4) | - | MD 30.51 higher  (11.56 lower to 72.57 higher) | - | 85 | ⨁◯◯◯ VERY LOW | Risk of bias (-1)^a^ Inconsistency (-1)^b^ Imprecision (-1)^c^ |
| mMRC | Total (≤4 weeks) | 200 (2) | - | MD 1.13 lower (1.21 to 1.05 lower) | - | 94 | ⨁⨁◯◯ LOW | Risk of bias (-1)^a^ Inconsistency (-1)^b^ |
| Subgroup 1 (COPD status) | Stable | 60 (1) | - | MD 0.28 lower (0.7 lower to 0.14 higher) | - | NA | ⨁⨁◯◯ LOW | Risk of bias (-1)^a^ Imprecision (-1)^c^ |
|  | AECOPD | 140 (1) | - | MD 1.16 lower (1.24 to 1.08 lower) | - | NA | ⨁⨁⨁⨁ HIGH | - |
| Frequency of acute exacerbation  (yr) | Total (stable) | 190 (2) | - | MD 0.6 lower (0.69 to 0.51 lower) | - | 0 | ⨁⨁⨁◯ MODERATE | Risk of bias (-1)^a^ |
| Subgroup 2 (Treatment duration) | ≤4 weeks | 60 (1) | - | MD 0.6 lower  (1.2 lower to 0 ) | - | NA | ⨁⨁◯◯ LOW | Risk of bias (-1)^a^ Imprecision (-1)^c^ |
|  | ≥2 months | 130 (1) | - | MD 0.6 lower  (0.69 to 0.51 lower) | - | NA | ⨁⨁⨁◯ MODERATE | Risk of bias (-1)^a^ |
| TER | Total | 1,024 (10) | 655 per 1,000 | 826 per 1,000 (727 to 943) | RR 1.26 (1.11 to 1.44) | 76 | ⨁◯◯◯ VERY LOW | Risk of bias (-1)^a^ Inconsistency (-1)^b^ Publication bias (-1)^d^ |
| Subgroup 1 (COPD status) | Stable | 539 (5) | 516 per 1,000 | 768 per 1,000 (557 to 1,000) | RR 1.49 (1.08 to 2.07) | 88 | ⨁⨁◯◯ LOW | Risk of bias (-1)^a^ Inconsistency (-1)^b^ |
|  | AECOPD | 285 (3) | 837 per 1,000 | 946 per 1,000 (879 to 1,000) | RR 1.13 (1.05 to 1.23) | 0 | ⨁⨁⨁⨁ HIGH | - |
|  | Unclear | 200 (2) | 710 per 1,000 | 873 per 1,000 (753 to 1,000) | RR 1.23 (1.06 to 1.42) | 0 | ⨁⨁⨁⨁ HIGH | - |
| Subgroup 2 (Treatment duration) | ≤4 weeks | 458 (6) | 783 per 1,000 | 908 per 1,000  (846 to 971) | RR 1.16  (1.08 to 1.24) | 0 | ⨁⨁⨁◯ MODERATE | Risk of bias (-1)^a^ |
|  | ≥2 months | 566 (4) | 534 per 1,000 | 827 per 1,000  (571 to 1,000) | RR 1.55  (1.07 to 2.25) | 91 | ⨁⨁⨁◯ MODERATE | Risk of bias (-1)^a^ |
| CAT | Total (≤4 weeks) | 373 (5) | - | MD 3.78 lower (5.73 to 1.83 lower) | - | 78 | ⨁⨁◯◯ LOW | Risk of bias (-1)^a^ Inconsistency (-1)^b^ |
| Subgroup 1 (COPD status) | Stable | 193 (3) | - | MD 3.15 lower (6.15 to 0.15 lower) | - | 76 | ⨁⨁◯◯ LOW | Risk of bias (-1)^a^ Inconsistency (-1)^b^ |
|  | AECOPD | 140 (1) | - | MD 6.17 lower (7.34 to 5 lower) | - | NA | ⨁⨁⨁⨁ HIGH | - |
|  | Unclear | 40 (1) | - | MD 2.21 lower (4.8 lower to 0.38 higher) | - | NA | ⨁⨁⨁◯ MODERATE | Imprecision (-1)^c^ |
| SGRQ | Total | 833 (6) | - | MD 7.56 lower (14.4 to 0.72 lower) | - | 93 | ⨁⨁◯◯ LOW | Risk of bias (-1)^a^ Inconsistency (-1)^b^ |
| Subgroup 1 (COPD status) | Stable | 608 (4) | - | MD 5.25 lower (10.88 lower to 0.37 higher) | - | 82 | ⨁◯◯◯ VERY LOW | Risk of bias (-1)^a^ Inconsistency (-1)^b^ Imprecision (-1)^c^ |
|  | AECOPD | 65 (1) | - | MD 3.26 lower (7.26 lower to 0.74 higher) | - | NA | ⨁⨁⨁◯ MODERATE | Imprecision (-1)^c^ |
|  | Unclear | 160 (1) | - | MD 21.17 lower (24.62 to 17.72 lower) | - | NA | ⨁⨁⨁⨁ HIGH | - |
| Subgroup 2 (Treatment duration) | ≤4 weeks | 65 (1) | - | MD 3.26 lower (7.26 lower to 0.74 higher) | - | NA | ⨁⨁⨁◯ MODERATE | Imprecision (-1)^c^ |
|  | ≥2 months | 768 (5) | - | MD 8.42 lower  (16.24 to 0.6 lower) | - | 94 | ⨁⨁⨁◯ MODERATE | Risk of bias (-1)^a^ |
| WHOQOL-BREF  (psychological) | Total | 327 (3) | - | MD 0.99 higher (0.35 to 1.64 higher) | - | 66 | ⨁⨁◯◯ LOW | Risk of bias (-1)^a^ Inconsistency (-1)^b^ |
| Subgroup (COPD status/Treatment duration) | Stable/≥2 months | 262 (2) | - | MD 1.46 lower (3.63 lower to 0.7 higher) | - | 0 | ⨁⨁◯◯ LOW | Risk of bias (-1)^a^ Imprecision (-1)^c^ |
|  | AECOPD/≤4 weeks | 65 (1) | - | MD 1.23 higher (0.56 to 1.9 higher) | - | NA | ⨁⨁⨁⨁ HIGH | - |
| WHOQOL-BREF  (physical health) | Total (AECOPD/≤4 weeks) | 65 (1) | - | MD 0.46 higher (0.23 lower to 1.15 higher) | - | NA | ⨁⨁⨁◯ MODERATE | Imprecision (-1)^c^ |
| WHOQOL-BREF  (social relationships) | Total (AECOPD/≤4 weeks) | 65 (1) | - | MD 0.83 lower (1.55 to 0.11 lower) | - | NA | ⨁⨁⨁⨁ HIGH | - |
| WHOQOL-BREF  (environment) | Total (AECOPD/≤4 weeks) | 65 (1) | - | MD 0.04 lower (0.72 lower to 0.64 higher) | - | NA | ⨁⨁⨁◯ MODERATE | Imprecision (-1)^c^ |
| Adverse events | Total | 1,045 (8) | 23 per 1,000 | 25 per 1,000 (12 to 53) | RR 1.08 (0.51 to 2.28) | 22 | ⨁⨁◯◯ LOW | Risk of bias (-1)^a^ Imprecision (-1)^c^ |
| Subgroup 1 (COPD status) | Stable | 641 (6) | 34 per 1,000 | 24 per 1,000 (10 to 59) | RR 0.72 (0.29 to 1.77) | 0 | ⨁⨁◯◯ LOW | Risk of bias (-1)^a^ Imprecision (-1)^c^ |
|  | AECOPD | 244 (1) | 16 per 1,000 | 49 per 1,000 (10 to 239) | RR 3.00 (0.62 to 14.57) | NA | ⨁⨁⨁◯ MODERATE | Imprecision (-1)^c^ |
|  | Unclear | 160 (1) | 0 per 1,000 | 0 per 1,000 (0 to 0) | not estimable | NA | ⨁⨁⨁⨁ HIGH | - |
| Subgroup 2 (Treatment duration) | ≤4 weeks | 437 (4) | 23 per 1,000 | 39 per 1,000  (14 to 110) | RR 1.71  (0.61 to 4.79) | 37 | ⨁⨁◯◯ LOW | Risk of bias (-1)^a^ Imprecision (-1)^c^ |
|  | ≥2 months | 608 (4) | 24 per 1,000 | 14 per 1,000  (5 to 45) | RR 0.59  (0.19 to 1.89) | 0 | ⨁⨁◯◯ LOW | Risk of bias (-1)^a^ Imprecision (-1)^c^ |

**Abbreviations.** AECOPD, acute exacerbations of chronic obstructive pulmonary disease; CAT, chronic obstructive pulmonary disease assessment test; CI, confidence interval; COPD, chronic obstructive pulmonary disease; FEV1, forced expiratory volume in one second; FVC, forced vital capacity; GRADE, grading of recommendations assessment, development, and evaluation; MD, mean difference; mMRC, modified medical research council dyspnea scale; NA, not applicable; RCT, randomized controlled trial; RR, risk ratio; SGRQ, St. George respiratory questionnaire; TER, total effective rate; VC, vital capacity; WHOQOL-BREF, World Health Organization quality of life instruments-abbreviated version; 6MWD, 6-minute walking distance.

^a^ high risk of performance, attrition, or reporting bias; ^b^ The direction of the effect is different and *I^2^* ≥ 50%; ^c^ wide confidence interval; ^d^ funnel plot asymmetry.
