# Supplementary material for: Herbal Medicine Compared to Placebo for Chronic Obstructive Pulmonary Disease: A Systematic Review and Meta-Analysis
Source: Front Pharmacol. 2021 Oct 20;12:717570. doi: 10.3389/fphar.2021.717570 (PMC8564496; doi:10.3389/fphar.2021.717570)
Supplement: Supplementary file 6 [file Table6.DOCX]

**Supplement 3. Excluded studies after full-text review**

**I. Not placebo control**

1. Qian CL, Fan R. Effect of Pingchuan Guben decoction on patients with chronic obstructive pulmonary disease: Results from a randomized comparative effectiveness research trial. Experimental and Therapeutic Medicine. 2017;14(4):3915-25.

**II. Conference proceeding without detailed research data**

1. Sun Z, Guo S, Liu E, Feng J, Fu M, Lian F, et al. The effect of a traditional chinese drug on chronic obstructive pulmonary disease stable phase. American Journal of Respiratory and Critical Care Medicine. 2012;185.

**III. Secondary analysis of RCT**

1. 赵丹. 调补肺肾法治疗慢阻肺稳定期临床疗效评价指标体系研究 [博士]: 北京中医药大学; 2011.

**IV. Using single herb**

1. Criner GJ, Dransfield MT, Arora S, Rogers TJ, Pritchard J, Engel C, et al. A randomized, double-blind, placebo controlled, multicenter phase 2a study to assess safety, daily respiratory symptoms, pharmacokinetics, and biomarker variations after administration of either YPL-001, or placebo in patients with moderate to severe chronic obstructive pulmonary disease. American journal of respiratory and critical care medicine. 2018;197(MeetingAbstracts).

**V. Not describing the homogeneity of herbal medicine and placebo**

**V-I. For stable COPD**

1. Hong M, Hong C, Chen H, Ke G, Huang J, Huang X, et al. Effects of the Chinese herb formula Yufeining on stable chronic obstructive pulmonary disease A randomized, double-blind, placebo-controlled trial. Medicine (United States). 2018;97(39).

2. Jiang F, Yan Y, Yang L, Song Q, Li Y. Impact of Chinese herb on quality of life of stable chronic obstructive pulmonary disease: A randomized controlled study. Zhongguo Zhongyao Zazhi. 2011;36(22):3203-6.

3. Li FS, Zhang YL, Li Z, Xu D, Liao CY, Ma H, et al. Randomized, double-blind, placebo-controlled superiority trial of the Yiqigubiao pill for the treatment of patients with chronic obstructive pulmonary disease at a stable stage. Exp Ther Med. 2016;12(4):2477-88.

4. Li SY, Zhou QW, Wang MH, Wang HF, Wu JZ. Effect of bufei yishen granule on pulmonary ventilation function and immunological function of patients with chronic obstructive pulmonary disease in remission phase: A randomized grouping and placebo controlled study. Chinese Journal of Clinical Rehabilitation. 2006;10(7):145-7.

5. 柯晓霞. 肺脾相关理论在慢性阻塞性肺疾病稳定期的临床应用研究 [硕士]: 广州中医药大学; 2010.

6. 江芳超, 阎明, 杨璐, 宋芊, 李友林. 中药治疗对COPD稳定期生命质量影响的多中心随机对照研究. 中国中药杂志. 2011;36(22):3203-6.

7. 桂吟哲. 肺肾同调治疗COPD稳定期的系统评价及临床研究 [博士]: 广州中医药大学; 2014.

8. 顾文超, 袁亚平, 杨华, 吴浩, 王林宣. 健脾补肾化痰方在稳定期慢性阻塞性肺疾病中的作用机制探讨. 世界临床药物. 2018;39(8):535-40.

9. 金晶, 王晶, 姚梓平, 李风森. 益气固表丸对COPD稳定期患者Th17/Treg平衡的影响. 新疆医科大学学报. 2018;41(09):1182-5+9.

10. 耑冰, 胡天成, 陈绍平, 蔡定筠, 刘小燕. 六君子汤对COPD稳定期氧化/抗氧化失衡的影响. 中医药临床杂志. 2006;18(3):262-3.

11. 杜亚君, 刘恩顺, 孙增涛, 付敏, 封继宏. 补肺颗粒对慢性阻塞性肺疾病稳定期患者生活质量与肺功能的影响. 新中医. 2013;45(5):37-9.

12. 刘恩顺, 孙增涛, 付敏, 封继宏, 王强, 关鹏, editors. 补肺颗粒治疗130例COPD稳定期患者随机、安慰剂对照临床研究. 全国中医内科肺系病第十四次学术研讨会; 2010; 中国内蒙古海拉尔.

13. 刘恩顺, 孙增涛, 付敏, 封继宏, 王强, 关鹏, editors. 补肺颗粒治疗慢性阻塞性肺疾病稳定期患者随机、安慰剂对照临床研究. “新成果·新进展·新突破”中华中医药学会2013年学术年会、第三次中华中医药科技成果论坛; 2013; 中国北京.

14. 刘恩顺, 孙增涛, 付敏, 封继宏, 王强, 关鹏. 补肺颗粒治疗慢性阻塞性肺疾病稳定期患者随机、安慰剂对照临床研究. 中华中医药学会2013年学术年会论文集; 北京2013. p. 173-7.

15. 刘萍. 补肺一号方减少慢性阻塞性肺疾病肺气虚证急性加重风险的临床研究 [硕士]: 福建中医药大学; 2015.

16. 刘学强, 谢文英. 平喘益气颗粒治疗老年稳定型慢性阻塞性肺疾病58例. 河南中医. 2015;35(8):1997-8.

17. 李乔, 卢家胜, 班文明, 赵卫星, 陆岗, 张飞虎, et al. 自拟补肺合剂对COPD稳定期患者肺功能的影响. 中医药临床杂志. 2016;28(7):962-5.

18. 李素云. 以补肺益肾法复方制剂调节慢性阻塞性肺疾病缓解期患者肺通气及免疫功能：随机分组安慰剂对照. 中国临床康复. 2006;10(7).

19. 李素云, 李建生, 马利军, 周庆伟, 李成海. 补肺益肾颗粒对COPD缓解期患者细胞外基质的影响. 中国中医药信息杂志. 2003;10(9):13-5.

20. 李素云, 周庆伟, 吴纪珍. 补肺益肾颗粒对COPD缓解期患者肺通气功能和免疫功能的影响. 山东中医杂志. 2003;22(6):333-5.

21. 李素云, 周庆伟, 王明航, 王海峰, 吴纪珍. 以补肺益肾法复方制剂调节慢性阻塞性肺疾病缓解期患者肺通气及免疫功能:随机分组安慰剂对照. 中国临床康复. 2006;10(7):145-7.

22. 李风森, 徐丹, 高振, 李争, 荆晶, 张艳丽, et al. 益气固表丸联合平喘敷贴膏治疗慢性阻塞性肺疾病稳定期临床研究. 中国中医药信息杂志. 2017;24(6):22-6.

23. 李兴文, 戴路明, 黄维丽, 方利洲, 杨玉萍, 张剑青. 云南白药对稳定期COPD痰液细胞因子调控的研究. 昆明医学院学报. 2001;22(2):29-31,7.

24. 李兴文, 杨玉萍, 方利洲, 戴路明, 黄维丽. 云南白药对稳定期慢性阻塞性肺疾病痰液炎性细胞调控研究. 中国新药杂志. 2001;10(4):282-4.

25. 丰银平. 基于"金水相生"理论研究百合固金汤加减对COPD稳定期的疗效观察. 第三届民族传统医学与现代医学国际学术大会暨第十三次全国中西医结合防治呼吸系统疾病学术研讨会论文集; 西宁2014. p. 275-8.

26. 付敏, 刘恩顺, 孙增涛. 补肺颗粒治疗COPD稳定期60例临床观察. 光明中医. 2009;24(3):455-6.

27. 付敏, 孙增涛, 刘恩顺, 封继宏, 杨晓. 补肺颗粒对稳定期COPD患者肺功能的影响. 上海中医药杂志. 2012;46(1):37-8.

28. 付敏, 孙增涛, 刘恩顺, 封继宏, 杨晓. 补肺颗粒治疗COPD稳定期临床研究. 中华中医药学刊. 2012;30(8):1735-7.

29. 付敏, 孙增涛, 刘恩顺, 封继宏, 王强. 补肺颗粒治疗130例COPD稳定期患者随机、安慰剂对照临床研究. 全国中医药博士生创新发展学术论坛论文集; 北京2011. p. 230-5.

30. 付敏, 孙增涛, 刘恩顺, 封继宏, 王强. 补肺颗粒对COPD稳定期患者血清IL-6水平的影响. 世界中西医结合杂志. 2012;07(6):529-31.

31. 付敏, 孙增涛, 刘恩顺, 封继宏, 王强, 关鹏, editors. 补肺颗粒治疗130例COPD稳定期患者随机、安慰剂对照临床研究. 第十一次全国中西医结合防治呼吸系统疾病学术研讨会; 2010; 中国北京.

32. 山萌, 黄刚, 李猛, 王志梅, 蔡绪明, 杨栓柱, et al. 益肺化痰活血颗粒治疗慢阻肺稳定期气虚痰瘀互结型的临床研究. 现代中西医结合杂志. 2020;29(23):2527-30,44.

33. 孙杰, 韩敏娟, 张旭辉, 曹鹏鹏. 咳喘宁胶囊对慢性阻塞性肺疾病稳定期患者气道炎症细胞及炎性介质的影响. 中国中医药信息杂志. 2014(9):21-3,4.

34. 孙杰, 韩敏娟, 张旭辉, 曹鹏鹏. 咳喘宁胶囊治疗稳定期慢性阻塞性肺疾病随机对照试验. 中国中医药信息杂志. 2014(7):22-4.

35. 孙增涛, 刘恩顺, 封继宏, 付敏. 补肺颗粒对COPD稳定期患者生活质量的影响. 上海中医药杂志. 2009;43(3):15-6.

36. 孙增涛, 刘恩顺, 封继宏, 付敏. 补肺颗粒治疗慢性阻塞性肺疾病稳定期临床疗效评价. 新中医. 2009;41(3):37-8.

37. 孙增涛, 付敏, 李月川, 吴琦, 刘恩顺, 封继宏. 补肺颗粒对慢性阻塞性肺疾病稳定期患者生活质量的影响. 中医杂志. 2012;53(11):930-2.

38. 孙增涛, 付敏, 李月川, 吴琦, 刘恩顺, 封继宏. 中药合理干预对稳定期COPD患者生活质量的影响. 第四届世界中西医结合大会论文集; 天津2012. p. 259-60.

39. 杨珺超, 王真, 楼雅芳, 徐俭朴, 洪辉华, 陈瑞琳, et al. 益气健脾颗粒剂联合西药治疗慢性阻塞性肺疾病稳定期肺脾气虚型60例临床观察. 中医杂志. 2013;54(22):1933-6.

40. 杨珺超, 王真, 徐俭朴, 楼雅芳, 陈瑞琳, 俞宁宁, et al. 益气健脾法对144例慢性阻塞性肺疾病稳定期肺脾气虚型患者生活质量的影响. 中华中医药学会2013年学术年会论文集; 北京2013. p. 191-4.

41. 杨珺超, 王真, 徐俭朴, 楼雅芳, 陈瑞琳, 俞宁宁, et al. 益气健脾法对72例慢性阻塞性肺疾病稳定期肺脾气虚型患者生活质量的影响. 浙江省中西医结合学会呼吸病专业委员会第九次学术年会论文集; 浙江丽水2013. p. 73-7.

42. 杨珺超, 王真, 徐俭朴, 楼雅芳, 陈瑞琳, 俞宁宁, et al. 益气健脾法对72例慢性阻塞性肺疾病稳定期肺脾气虚型患者生活质量的影响. 中华中医药学会第十七次全国中医肺系病学术交流会论文集; 烟台2013. p. 82-5.

43. 杨珺超, 王真, 徐俭朴, 楼雅芳, 陈瑞琳, 俞宁宁, et al. 益气健脾法对144例慢性阻塞性肺疾病稳定期肺脾气虚型患者生活质量的影响. 中华中医药杂志. 2014;29(2):638-40.

44. 杨晓龙. 问卷评分和肺通气功能评价补肺颗粒对稳定期慢阻肺的疗效. 中成药. 2015;37(10):2146-9.

45. 吴标. 加味参蛤散对慢阻肺缓解期患者肺通气功能临床观察. 医药产业资讯. 2006;3(3):70.

46. 王斌灿, 陈志斌, 王春娥, 刘萍. 补肺一号方治疗慢性阻塞性肺疾病稳定期肺气虚证的临床研究. 广西中医药. 2016;39(4):7-10.

47. 王真, 杨珺超, 楼雅芳, 徐俭朴, 洪辉华, 陈瑞琳, et al., editors. 益气健脾法对144例慢性阻塞性肺疾病稳定期肺脾气虚型患者的临床疗效观察. 中华医学会呼吸病学年会——2013（第十四次全国呼吸病学学术会议）; 2013; 中国辽宁大连.

48. 王肖艳, 王俊月, 宋倩红, 牛贺, 谢文英, 包永生. 二陈汤加味方对COPD稳定期患者血清中MMP-9、TIMP-1活性及其比值的影响. 中国老年学杂志. 2020;40(18):3868-71.

49. 张杰良, 郑世良, 刘美娟, 王诚, 李延玲. 三子止咳胶囊对 COPD 稳定期患者肺功能的改善作用及机制. 山东医药. 2016;56(21):22-4.

50. 张高, 孙志佳, 褚庆民. 补肺健脾益肾法对早期慢性阻塞性肺疾病稳定期干预作用. 辽宁中医药大学学报. 2015;17(12):78-81.

51. 张双胜, 程荣健, 程道胜, 陈虹心. 健脾补肺法和固肾益肺法对稳定期慢性阻塞性肺疾病患者营养状况、TNF-α及肺功能影响的临床研究. 中国中医药科技. 2007;14(2):70-1.

52. 张旭辉. 咳喘宁胶囊治疗稳定期慢性阻塞性肺疾病患者的疗效评价. 甘肃中医药大学附属医院第十一届学术年会论文集; 兰州2015. p. 257-61.

53. 张旭辉, 杨虹. 咳喘宁胶囊治疗稳定期慢性阻塞性肺疾病的疗效评价. 西部中医药. 2016;29(3):1-4.

54. 章潜. 益气健脾颗粒对COPD稳定期患者肺功能指标的影响. 浙江中医药大学学报. 2014;38(5):575-7.

55. 张会哲, 张桂才, 蔡元培, 成向进. 补肺颗粒对稳定期慢性阻塞性肺疾病患者血清T3、T4及COS的影响. 湖南中医杂志. 2017;33(2):43-5.

56. 郑硕锋. 基于形神一体观的COPD稳定期辨证论治临床疗效评价的探索研究 [硕士]: 北京中医药大学; 2010.

57. 赵亚玲, 宋鸿儒, 费晋秀, 王娟, 胡平, 王荣, et al. 山脾合剂对高龄稳定期COPD患者BODE指数和生活质量的影响. 陕西中医. 2012;33(4):387-9.

58. 赵亚玲, 宋鸿儒, 费晋秀, 张兵华, 刘秋平. 健脾补肾中药对稳定期中重度慢性阻塞性肺疾病营养状态和运动能力的影响. 中华临床医师杂志(电子版). 2012;06(5):1336-8.

59. 赵亚玲, 宋鸿儒, 张兵华, 王荣, 周伟. 健脾补肾对高龄稳定期COPD患者营养状况和体液免疫影响. 西北国防医学杂志. 2012;33(3):242-4.

60. 赵亚玲, 张志惠, editors. 健脾补肾中药对稳定期中重度慢性阻塞性肺病BODE和活质量的影响. 中华医学会呼吸病学年会——2013（第十四次全国呼吸病学学术会议）; 2013; 中国辽宁大连.

61. 赵亚玲, 张志惠. 健脾补肾中药对稳定期中重度慢性阻塞性肺病BODE和活质量的影响. 第六届全国慢性阻塞性肺疾病学术会议论文集; 银川2013. p. 1-12.

62. 赵亚玲, 张志惠. 健脾补肾中药对中重度慢阻肺稳定期患者BODE指数和生活质量的影响. 第六届全国慢性阻塞性肺疾病学术会议论文集; 银川2013. p. 8-9.

63. 周庆伟. 缓肺颗粒对慢阻肺缓解期患者免疫功能的影响. 辽宁中医杂志. 2005;32(7):670-1.

64. 周庆伟, 李素云, 吴纪珍. 缓肺颗粒对慢性阻塞性肺疾病缓解期患者肺通气功能的影响. 中国中西医结合杂志. 2005;25(7):649.

65. 曾林生. 补肺健脾益肾法治疗早期COPD稳定期的疗效观察 [硕士]: 广州中医药大学; 2013.

66. 漆冬梅. 培土生金法治疗慢性阻塞性肺疾病稳定期临床疗效观察 [硕士]: 广州中医药大学; 2009.

67. 何迎春, 陈海玲, 张如富. 培土生金法改善慢性阻塞性肺病稳定期患者生活质量的临床疗效观察. 光明中医. 2010;25(5):776-7.

68. 何迎春, 陈海玲, 张如富. 补中益气汤加减改善慢性阻塞性肺病稳定期患者生活质量的临床疗效观察. 中华中医药学刊. 2010;28(3):506-7.

69. 韩敏娟. 咳喘宁胶囊对稳定期慢性阻塞性肺疾病患者气道炎症影响及疗效评价 [硕士]: 甘肃中医学院; 2015.

70. 韩萍. 清肺调血汤合金匮肾气丸治疗COPD稳定期20例临床观察. 光明中医. 2011;26(7):1368-9.

71. 许洁翎, 肖波, 李慧. 加味半夏泻心汤治疗慢性阻塞性肺疾病稳定期临床研究. 陕西中医. 2018;39(3):295-7.

72. 洪敏俐, 杨朝阳, 柯庚申, 洪春霖, 刘燕鸿, 黄小华, et al. 愈肺宁方对慢性阻塞性肺疾病稳定期患者中医证素的影响. 中华中医药杂志. 2015;30(05):1666-9.

73. 洪春霖, 洪敏俐, 陈慧暖, 柯庚申, 黄小华, 刘燕鸿, et al. 愈肺宁方对慢性阻塞性肺疾病稳定期患者诱导痰炎症细胞和LTB_4的影响. 世界科学技术-中医药现代化. 2015;17(12):2517-21.

74. 洪辉华, 王真, 杨珺超, 楼雅芳, 徐俭朴, 周林水, et al. 益气健脾法治疗慢性阻塞性肺疾病稳定期肺脾气虚型患者的多中心临床研究. 中华中医药学会第十七次全国中医肺系病学术交流会论文集; 烟台2013. p. 145-9.

75. 洪辉华, 王真, 杨珺超, 楼雅芳, 徐俭朴, 周林水, et al. 益气健脾法治疗慢性阻塞性肺疾病稳定期肺脾气虚型患者的多中心临床研究. 浙江省中西医结合学会呼吸病专业委员会第九次学术年会论文集; 浙江丽水2013. p. 55-60.

76. 洪辉华, 王真, 杨珺超, 楼雅芳, 徐俭朴, 周林水, et al. 益气健脾法治疗慢性阻塞性肺疾病稳定期肺脾气虚型患者的多中心临床研究. 中华中医药杂志. 2014;29(4):1153-6.

77. 黄刚. 益肺化痰活血汤治疗慢阻肺稳定期气虚痰瘀互结型的临床观察 [硕士]: 陕西中医药大学; 2018.

78. 黄青松, 唐文君, 孙群. 健脾补肾法对慢性阻塞性肺疾病稳定期中IL-8与TNF-α的变化及意义. 成都中医药大学学报. 2015;38(4):34-8.

79. 黄平富, 王胜, 王浩, 赵志奋, 李泽庚, 胡国俊. 金龙蛤蚧平喘胶囊治疗稳定期慢性阻塞性肺疾病临床观察. 安徽中医药大学学报. 2016;35(04):41-6.

**V-II. For AECOPD**

80. Jiansheng L, Haifeng W, Suyun L, Hailong Z, Xueqing Y, Xiaoyun Z, et al. Effect of sequential treatment with TCM syndrome differentiation on acute exacerbation of chronic obstructive pulmonary disease and AECOPD risk window. Complement Ther Med. 2016;29:109-15.

81. Jinfang M, Jinping Z, Nanshan Z, Chunxue B, Haoyan W, Juan D, et al. Effects of YuPingFeng granules on acute exacerbations of COPD: a randomized, placebo-controlled study. International journal of chronic obstructive pulmonary disease. 2018;13.

82. Li J, Zhang H, Ruan H, Si Y, Sun Z, Liu H, et al. Effects of Chinese Herbal Medicine on Acute Exacerbations of COPD: A Randomized, Placebo-Controlled Study. Int J Chron Obstruct Pulmon Dis. 2020;15:2901-12.

83. Zhen G, Jing J, Dan X, Zheng L, Fengsen L, Qi S. A Randomized Controlled Study of the Yi Qi Gu Biao Pill in the Treatment of Frequent Exacerbator Phenotype in Chronic Obstructive Pulmonary Disease (Lung and Spleen Qi Deficiency Syndrome). Evidence-based Complementary and Alternative Medicine. 2017;2017.

84. 高培阳, 周平, 张川, 钟兴美, 肖先华, 张松, et al. 中西医结合综合治疗脾肾阳虚和痰湿内蕴证慢性阻塞性肺疾病急性加重合并呼吸衰竭患者的临床研究. 中国中西医结合急救杂志. 2014(4):245-8.

85. 李建生, 李素云, 陈萍, 李彬, 马利军, 于洪涛, et al. 燥湿化痰方药治疗慢性阻塞性肺疾病急性加重期痰湿壅肺证的临床疗效评价. 辽宁中医杂志. 2010;37(9):1721-5.

86. 李博. 止咳平喘软胶囊治疗慢性支气管炎急性发作期的临床观察及对血清IL-6的影响 [硕士]: 黑龙江中医药大学; 2011.

87. 付士民. 化痰汤治疗COPD急性加重期证属痰浊阻肺患者的疗效. 中国医学创新. 2010;07(33):44-6.

88. 尚立芝, 季书, 谢文英, 薛红莉, 刘志勇, 刘坦, et al. 二陈汤加味对COPD急性期患者CC16,SP-D及HAT/HDAC的影响. 中国实验方剂学杂志. 2017;23(10):163-70.

89. 王德琴, 徐彬, 李泽庚, 彭波, 童佳兵, 杨程. 芪白平肺胶囊治疗痰瘀阻肺型COPD临床观察. 中华中医药学会肺系病分会成立大会暨第十五次全国中医肺系病学术交流大会论文集; 北京2011. p. 177-8.

90. 王德琴, 徐彬, 李泽庚, 彭波, 童佳兵, 杨程. 芪白平肺胶囊治疗痰瘀阻肺型COPD临床观察. 中医药临床杂志. 2012;24(4):301-2.

91. 王传博, 李泽庚, 彭波, 童佳兵, 杨程. 芪白平肺胶囊对慢阻肺患者圣乔治呼吸问卷积分的影响. 中华中医药学刊. 2012;30(2):266-8.

92. 周佳佳, 林呼, 古威. 清金化痰汤合麻杏石甘汤治疗COPD急性加重期痰热郁肺证型临床观察. 陕西中医. 2017;38(11):1532-3.

93. 秦凤霞, 倪海滨, 黄小菲, 赵慧, 魏凤琴. 肺肠合治法对慢性阻塞性肺疾病急性加重患者炎症水平的影响. 中国医药指南. 2019;17(32):179-80.

94. 邹新中. 芪味理肺汤联合沙美特罗替卡松治疗老年AECOPD的疗效评价. 中国地方病防治杂志. 2016;31(10):1192-3.

**V-III. For unclear COPD**

95. Qu NN, Liu H, Ma LJ, Zhao KM, Zheng X, Shu P. Compound Yiqi Wenyang Huoxue Formula in treatment of qi deficiency and blood stasis combining phlegm obstruction type of chronic obstructive pulmonary disease. Liaoning journal of traditional chinese medicine [liao ning zhong yi za zhi]. 2015;42(8):1438‐40.

96. Zhao YL, Song HR, Fei JX, Liang Y, Zhang BH, Liu QP, et al. The effects of Chinese Yam-Epimedium mixture on respiratory function and quality of life in patients with chronic obstructive pulmonary disease. Journal of Traditional Chinese Medicine. 2012;32(2):203-7.

97. 曲妮妮, 刘浩, 马丽佳, 赵克明, 郑忻, 束沛. 益气温阳活血中药干预慢性阻塞性肺疾病肺动脉高压机理探讨. 中华中医药学刊. 2015;33(5):1218-20.

98. 曲妮妮, 刘浩, 马丽佳, 赵克明, 郑忻, 束沛. 益气温阳活血中药复方治疗慢性阻塞性肺疾病气虚血瘀兼痰阻患者临床观察. 辽宁中医杂志. 2015;42(8):1438-40.

99. 曲妮妮, 庞立健, 刘浩, 束沛, 石晓乐, 项继静, et al. 益气温阳活血中药对慢性阻塞性肺疾病患者生存质量影响的研究. 合肥2014. p. 269-72.

100. 郭思佳, 孙增涛, 李月川, 吴琦, 刘恩顺, 封继宏, et al. 补肺颗粒对慢性阻塞性肺疾病稳定期患者血清IL-6,IL-8,TNF-α及TGF-β1水平的影响. 时珍国医国药. 2013;24(12):2933-4.

101. 郭思佳, 孙增涛, 李月川, 吴琦, 封继宏, 窦钊. 补肺颗粒对轻中度慢性阻塞性肺疾病稳定期患者血清IL-33/sST2轴及相关炎性因子表达的影响:多中心、双盲、随机对照试验. 中国中西医结合杂志. 2018;38(9):1034-9.

102. 邱晓青. 补肺固本汤对慢性阻塞性肺炎患者肺通气功能的影响. 医学信息（上旬刊）. 2011;24(10):6561-2.

103. 童佳兵, 王传博, 彭波, 杨程, 李泽庚, 王浩. 芪白平肺胶囊对慢性阻塞性肺疾病痰瘀阻肺证患者中医证候积分的影响. 中外健康文摘. 2011;08(18):51-3.

104. 童佳兵, 王传博, 彭波, 杨程, 李泽庚, 王浩. 芪白平肺胶囊治疗慢性阻塞性肺疾病痰瘀阻肺证疗效分析. 中华中医药学刊. 2012;30(1):44-5.

105. 罗春凤, 陈婉, 赵丽红, 张容. 纳气通络方联合西药治疗肺肾气虚型慢性阻塞性肺疾病稳定期临床研究. 上海中医药杂志. 2013;47(9):41-3.

106. 刘冀. 玉屏风散对老年慢性阻塞性肺病患者免疫功能的影响. 四川中医. 2012;30(4):82-3.

107. 李云鹏. 清肺止咳治疗中医咳嗽风邪化热壅肺证的临床研究 [硕士]: 湖北中医药大学;湖北中医学院; 2009.

108. 李倩男, 房颖, 王海英. 化痰通络益气养阴法治疗慢性阻塞性肺疾病合并肺间质纤维化疗效观察. 现代中西医结合杂志. 2019;28(21):2338-41.

109. 李泽庚, 王传博, 彭波, 童佳兵, 杨程. 芪白平肺胶囊对慢性阻塞性肺疾病患者BODE指数的影响. 中医杂志. 2010;51(11):987-9.

110. 李泽庚, 王传博, 彭波, 童佳兵, 杨程, 刘志刚. 芪白平肺胶囊对慢阻肺患者圣乔治呼吸问卷积分的影响. 第十一次全国中西医结合防治呼吸系统疾病学术研讨会论文集; 北京2010. p. 173-7.

111. 李泽庚, 王传博, 彭波, 童佳兵, 杨程, 张念志, et al. 芪白平肺胶囊对慢性阻塞性肺疾病患者BODE指数的影响. 全国中医内科肺病第十四次学术研讨会论文集; 海拉尔2010. p. 15-9.

112. 李泽庚, 王传博, 彭波, 杨程, 童佳兵, 王浩. 芪白平肺胶囊对慢阻肺痰瘀阻肺证患者中医证候积分的影响. 中华中医药学会肺系病分会成立大会暨第十五次全国中医肺系病学术交流大会论文集; 北京2011. p. 164-7.

113. 李风森, 徐丹, 高振, 李争, 荆晶, 张艳丽, et al. 益气固表丸联合平喘贴敷膏治疗稳定期慢性阻塞性肺病(肺脾气虚型)的短期疗效观察. 第十四次全国中西医结合防治呼吸系统疾病学术研讨会论文集; 贵州兴义2016. p. 511-21.

114. 牟德辉, 何江, 李溥, 管慧, 周锦勇. 天人平喘胶囊治疗慢性阻塞性肺疾病疗效评价. 基层医学论坛. 2013(28):3724-5.

115. 时婧. 中药黄金开肺汤结合头孢他啶治疗 慢性阻塞性肺炎124例分析. 中外女性健康研究. 2019(19):52-3.

116. 蔡柏蔷, 朱元珏, 徐凌, 张弘, 郭子健, 马毅, et al. 化瘀咳喘片(814)在慢性阻塞性肺疾病中的治疗作用(312例临床治疗分析). 医学研究通讯. 2002;31(4):14-8.

117. 何江, 常智玲, 管慧, 周棉勇, 李世红, 廖沙, et al. 天人平喘胶囊治疗慢性阻塞性肺病(肺肾气虚型)随机双盲对照研究. 中国实验方剂学杂志. 2012;18(14):250-3.

118. 黄大文, 陈鹏, 李英姿, 孔凡平, 邱勇. 补肺固本冲剂对慢性阻塞性肺疾患肺通气功能的影响. 中国中医药信息杂志. 2002;9(10):17-8.

**V-IV. For chronic bronchitis**

119. Mao B, Zhang RM, Li TQ. Clinical observation on effect of kesuning granule in treating acute onset of chronic bronchitis. Zhongguo zhong xi yi jie he za zhi zhongguo zhongxiyi jiehe zazhi = chinese journal of integrated traditional and western medicine. 2002;22(8):597‐8.

120. 德吉措. 藏医六味藏红花丸对治疗慢性支气管炎疾病患者的治疗效果比较. 健康大视野. 2019(13):85,4.

121. 杜琳麟. 止咳平喘软胶囊治疗慢性支气管炎急性发作期的临床观察及对血清IL-8的影响 [硕士]: 黑龙江中医药大学; 2011.

122. 李朝辉. 清肺止咳口服液治疗慢性支气管炎急性发作的临床疗效. 当代医药论丛. 2014;12(01):180-1.

123. 熊新军. 清肺止咳口服液治疗慢性支气管炎急性发作（风邪化热壅肺型咳嗽）的临床研究 [硕士]: 湖北中医药大学;湖北中医学院; 2007.

124. 彭先祝. 止咳平喘软胶囊治疗慢性支气管炎急性发作期的临床观察及对血清TNF-α的影响 [硕士]: 黑龙江中医药大学; 2011.

**VI. Using duplicated data**

1. 吴蕾, 林琳, 许银姬, 孙志佳, 高雪, 惠萍, et al. 健脾益肺Ⅱ号治疗慢性阻塞性肺疾病稳定期178例临床研究. 中医杂志. 2011(17):1465-8.

**VII. Unavailable the full-text**

1. 封继宏, 孙增涛, 刘恩顺, 付敏. 以冬病夏治理论为指导治疗慢性阻塞性肺疾病稳定期的初步研究. 第十一届中国科协年会论文集; 重庆2009. p. 81-4.

2. 谢天. 补肺健脾益肾法治疗COPD稳定期临床疗效研究 [硕士]: 上海中医药大学; 2014.

3. 吴国伟, 傅声武, 张卫, 江涛, 姚智伟, 来旭明, editors. 通腑泻肺方治疗慢性阻塞性肺疾病急性加重(AECOPD)的临床观察. 中华医学会第二届重症心脏全国学术大会暨第三届西湖重症医学论坛、2015年浙江省重症医学学术年会; 2015; 中国浙江杭州.

4. 刘恩顺. 补肺颗粒与安慰剂对照治疗COPD稳定期疗效评价指标权重的对比分析. 湖南中医药大学学报. 2016;36(A01).

5. 肖鹏云, 辛大永. 通腑泻肺方结合西医常规疗法治疗痰热遏肺型慢性阻塞性肺疾病急性加重患者临床研究. 国际中医中药杂志. 2017;39(10):887-90.
